# The host phylogeny determines viral infectivity and replication across *Staphylococcus* host species

**DOI:** 10.1371/journal.ppat.1011433

**Published:** 2023-06-08

**Authors:** Sarah K. Walsh, Ryan M. Imrie, Marta Matuszewska, Gavin K. Paterson, Lucy A. Weinert, Jarrod D. Hadfield, Angus Buckling, Ben Longdon

**Affiliations:** 1 Centre for Ecology and Conservation; Faculty of Environment, Science, and Economy; Biosciences; University of Exeter; Cornwall; United Kingdom; 2 Environment and Sustainability Institute; University of Exeter; Cornwall; United Kingdom; 3 Department of Medicine; University of Cambridge; Cambridge; United Kingdom; 4 Royal (Dick) School of Veterinary Studies and the Roslin Institute; University of Edinburgh;Edinburgh; United Kingdom; 5 Department of Veterinary Medicine; University of Cambridge; Cambridge; United Kingdom; 6 Institute of Evolutionary Biology; The University of Edinburgh; Edinburgh; United Kingdom; University of Colorado Denver - Anschutz Medical Campus, UNITED STATES

## Abstract

Virus host shifts, where a virus transmits to and infects a novel host species, are a major source of emerging infectious disease. Genetic similarity between eukaryotic host species has been shown to be an important determinant of the outcome of virus host shifts, but it is unclear if this is the case for prokaryotes where anti-virus defences can be transmitted by horizontal gene transfer and evolve rapidly. Here, we measure the susceptibility of 64 strains of *Staphylococcaceae* bacteria (48 strains of *Staphylococcus aureus* and 16 non-*S*. *aureus* species spanning 2 genera) to the bacteriophage ISP, which is currently under investigation for use in phage therapy. Using three methods–plaque assays, optical density (OD) assays, and quantitative (q)PCR–we find that the host phylogeny explains a large proportion of the variation in susceptibility to ISP across the host panel. These patterns were consistent in models of only *S*. *aureus* strains and models with a single representative from each *Staphylococcaceae* species, suggesting that these phylogenetic effects are conserved both within and among host species. We find positive correlations between susceptibility assessed using OD and qPCR and variable correlations between plaque assays and either OD or qPCR, suggesting that plaque assays alone may be inadequate to assess host range. Furthermore, we demonstrate that the phylogenetic relationships between bacterial hosts can generally be used to predict the susceptibility of bacterial strains to phage infection when the susceptibility of closely related hosts is known, although this approach produced large prediction errors in multiple strains where phylogeny was uninformative. Together, our results demonstrate the ability of bacterial host evolutionary relatedness to explain differences in susceptibility to phage infection, with implications for the development of ISP both as a phage therapy treatment and as an experimental system for the study of virus host shifts.

## Author summary

Virus host shifts, where a virus jumps into a new host from another species, are a major source of emerging infectious diseases. Virus host shifts have been shown to be more likely between animals and plant species that are closely related. However, there is much to learn about how relatedness predicts susceptibility in bacterial hosts. Here, we used a panel of 64 *Staphylococcus* bacteria and a virus that infects them to investigate how the relationship between host species influences their susceptibility to infection. We find high variation in susceptibility to infection across the host panel and that most of that variation can be explained by the relationship between hosts. This effect is seen consistently using three methods for assessing susceptibility and can be seen both within and between species. Additionally, we find that the relationship between hosts allows us to predict the susceptibility of an unknown host with some accuracy, although there are instances where susceptibility cannot be predicted. Overall, this suggests that closely related hosts will show similar susceptibility to infection and that the relationship between bacterial hosts can be used to predict the susceptibility of an unknown host with limited success.

## Introduction

Host shifts, where a pathogen jumps into a novel host species and establishes onward transmission, are a major source of emerging infectious diseases. While host shifts can occur with many types of pathogen, viruses are the most prolific [1, 2]. Accordingly, viruses originating in non-human animals make up the majority of recently emerged human infections [3–5], including several human pandemics: HIV-1, which jumped into humans from chimpanzees [3, 6]; influenza A viruses, which commonly emerge from wild aquatic birds [7–10]; and most recently SARS-CoV-2, which likely transmitted into humans from a bat reservoir [11–13]. Given the scale and speed at which emerging viruses can impact host populations, understanding the underlying causes of virus host shifts has become a major goal of infectious disease research.

The evolutionary relationships between hosts are a key factor in determining the success of a pathogen following transmission to a novel host species, and several studies have investigated the ability of host evolutionary relatedness to explain variation in infection traits. These studies have shown that virulence tends to increase [14–18], and transmission rate [14, 19] and pathogen load [18, 20, 21] decrease with greater evolutionary distance between donor and recipient host species. These ‘distance effects’ have been seen in viruses [19–21], bacterial pathogens [22–24], fungi [25, 26], and nematodes [18], as well as in reconstructions of virus host shifts and cross species transmissions in nature [19, 27]. Additionally, closely related species may share similar levels of susceptibility independent of evolutionary distance to the natural host. These ‘clade effects’ create a patchwork of host clades across a phylogeny that vary in their susceptibility to a pathogen, and have been demonstrated in experimental infections of fruit flies [17, 21] and in pathogens that repeatedly jump between distantly related hosts in nature [28–32]. These effects can act concurrently to influence host shifts, with distance effects making successful pathogen shifts into closely related hosts more likely, and clade effects allowing for host shifts into more distantly related clades of susceptible hosts.

While most studies investigate the role of eukaryotic host phylogenies, few have examined the influence of the host phylogeny on susceptibility to infection in a prokaryotic system. Host phylogenetic effects follow the conventional wisdom that more closely related species share more similar phenotypes, and so present similar environments to invading pathogens. In bacteria, there are several mechanisms by which bacteriophage (viruses that infect bacteria; hereafter ‘phage’) resistance may be acquired in a phylogenetically intractable way, including horizontal gene transfer (HGT) [33], recombination [34], and the acquisition of prophages conferring resistance to further phage infection [35]. It was previously demonstrated that the transfer of plasmids between bacterial hosts is more likely between recipients with near identical genomes to the plasmid donor, but the likelihood of plasmid transfer was not correlated with genetic distance between donors and recipients at larger evolutionary distances [36]. As such, mobile genetic elements containing phage resistance genes may transmit between bacterial hosts in a way that does not segregate phylogenetically. Additionally, it has been demonstrated that evolution of the bacterial core genome is almost entirely driven by recombination, and thus more determined by the spatial structure of microbes rather than clonal inheritance [34]. Finally, the presence of prophage in bacterial genomes providing superinfection immunity to lytic phage has been characterised [37–41], including in *Staphylococcus* [42], meaning that prior infection with temperate bacteriophage could influence the pattern of susceptibility to lytic phage across the phylogeny [35, 43]. Together, we may expect these mechanisms to lead to weaker phylogenetic signal in virus susceptibility in bacterial hosts than that seen in animals where immunity is largely vertically transmitted.

Recent years have seen a resurgence in interest in the use of phage to treat bacterial infections due to the continuing emergence of antimicrobial resistance [44–47]. Phage present a promising alternative to traditional antimicrobials in that they are self-amplifying, self-limiting, and have proved effective in the treatment of drug-resistant bacterial infections such as methicillin-resistant *Staphylococcus aureus* (MRSA) [48–50] and *Pseudomonas aeruginosa* [50]. Two key considerations in the design of phage therapies are the host range of the phage–and so the range of bacterial strains or species it can be used to treat–and the efficiency of the phage in replicating and killing its bacterial host, which is linked to host susceptibility. Studies have shown that phage host range can vary from a single host species [51–55] to broad host range generalists [51, 53–59], although a consensus has yet to be reached on the most effective method for quantifying phage host range [60–62]. Additionally, studies investigating susceptibility to phage across bacterial isolates have shown variation in susceptibility at both the host strain and species level [56, 57, 59].

An ability to explain and predict variation in phage host range and susceptibility would be beneficial in the design of future therapies, allowing for the more efficient design of broad range phage cocktails with high efficacy against multiple pathogens. Recently, the evolutionary relationships between bacterial hosts were shown to explain some of the variation in susceptibility of a panel of *S*. *aureus* hosts to several staphylococcal phages when measured using plaque assays [63], suggesting that the structure of the host phylogeny may be a useful tool in predicting bacterial susceptibility to phage. It remains to be seen whether the host phylogeny can be a useful tool in explaining variation in bacterial susceptibility across broader phylogenetic scales (i.e., across bacterial species or genera), or when applied to different measures or components of bacterial susceptibility.

Here, we use a broad host range bacteriophage (Intravenous Staphylococcal Phage; ISP) and a panel of 64 *Staphylococcaceae* isolates (encompassing 2 genera and 17 *Staphylococcus* species) to investigate how patterns of bacterial susceptibility are influenced by the evolutionary relationships between hosts. ISP, a double stranded DNA virus in the family *Myoviridae*, is closely related to *Staphylococcus* phage G1 [64, 65] and has shown success in the treatment of antimicrobial resistant infections clinically [66]. ISP was isolated in the 1920s from an unknown source [65] and has a broad experimental host range within *S*. *aureus*, infecting 86% isolates tested in a previous study [67]. However, in the same study, ISP was unable to experimentally infect nine *S*. *haemolyticus* isolates, suggesting that this host range may be species-specific. Staphylococci are a well-established model for bacteria-phage interactions, with investigations into staphylococcal phages occurring since the 1910s. Lytic staphylococcal phages have demonstrated broad host ranges [52, 56, 68, 69], antibiofilm activity [68, 70], and vary in their efficacies against bacterial infection [71–73]. Several mechanisms that are important for the interaction between staphylococci and their phage have been characterised, including common cell surface receptors used for attachment [74–79], and host resistance mechanisms such as the overproduction of surface proteins to block adsorption [80–82], restriction modification systems [83–85], and CRISPR targeted degradation of phage DNA [86–89]. Despite this, much remains to be understood about the interactions between staphylococcal phages and their hosts. An improved understanding of the mechanisms that underpin bacteria-phage interactions may provide more general insights into virus-host interactions and their implications in the emergence of viral pathogens into novel populations.

In this study we demonstrate that ISP is a broad host range bacteriophage, able to infect both within and among species of *Staphylococcaceae*. Given that different methods of quantifying host range can give different estimations, we assessed the susceptibility of our bacterial host panel to ISP using three methods: plaque assays, optical density assays, and quantitative PCR. We observed considerable variation in susceptibility to ISP across the host panel and showed that a high proportion of the observed variation in susceptibility was attributable to the relationship between host species.

## Methods

### *Staphylococcaceae* isolates

This study made use of 64 strains of *Staphylococcaceae*, representing a broad phylogenetic and geographic range of hosts. These strains spanned 2 genera and 17 species that were estimated to have last shared a common ancestor ~122mya [90]. Multi-Locus Sequencing Typing (MLST) revealed that 5 Clonal Complexes (CC) of *S*. *aureus* were represented in this panel, including major complexes CC1 and CC8 (S1 Table).

Each *Staphylococcaceae* sample was streaked on a LB-agar Miller (Formedium) plate (1.5% agar) and incubated for 24 hours at 37°C. A single colony was isolated and used to inoculate 5mL of LB broth, which was incubated at 37°C, 180rpm for 24 hours. All isolates were stored in 25% glycerol at -80°C. When required, isolates were grown up by inoculating 5mL of high-salt LB Miller broth (Formedium) in a sterile 30mL glass universal with a scraping of the frozen culture and incubating at 37°C, 180rpm overnight. To ensure that any observed differences in susceptibility to ISP were not a function of host availability (i.e., differences in the concentration of bacterial hosts in solution), calibration curves using optical density (OD) and colony forming units (CFU)/mL were generated for each strain. Overnight cultures were then normalised by dilution in LB Miller broth to give similar host densities prior to their use in susceptibility assays.

### Phage preparation

An isolate of ISP was kindly provided by Jean-Paul Pirnay and Maya Merabishvili at the Queen Astrid Military Hospital (Brussels, Belgium). ISP was propagated on the *S*. *aureus* strain 13S44S9 (chosen for consistency with previous studies [65]; hereafter referred to as the ‘propagation host’) and extracted using chloroform: 1mL aliquots of the suspension were treated with 10% chloroform, vortexed for 1.5 minutes, then centrifuged for 1 minute at 18 x g. The supernatant containing the phage was aliquoted into sterile 2mL Eppendorf tubes, and the process repeated a second time to ensure the complete removal of bacterial hosts.

The number of infectious phage present was quantified using plaque assays. ISP was 10-fold serially diluted in 1X M9 buffer (Merck Life Sciences) from 10^0^ to 10^−8^. 100μL of overnight culture of 13S44S9 and 50μL of diluted phage were added to 5mL of LB-agar (0.5% agar), gently mixed, and poured over a 20mL LB-agar Miller plate. Plates were left to dry before being inverted and incubated at 37°C for 24 hours. Plaques were counted and plaque forming units (PFU)/mL determined based on the dilution with the highest number of discernible plaques. Each plaque assay was repeated at least 3 times to account for plating error.

### Assessing susceptibility using plaque assays

10-fold serial dilutions of ISP (initial concentration 1.6x10^8^ PFU/mL, determined by plaquing on 13S44S9) were prepared in 1X M9 buffer, ranging from 10^0^ to 10^−8^. Bacterial isolates were diluted in LB Miller broth, plated out to a final density of 2x10^10^ bacteria/mL, and left to dry before 5μL of each ISP dilution was spotted on each LB-agar Miller plate. The plates were then inverted and incubated at 37°C. Plaques were counted after 24 hours and converted to PFU/μL, based on the dilution with the highest number of discernible plaques. Six biological replicates of the plaque assay were performed for each bacteria-phage pairing. As the range of PFU/mL across technical replicates in preliminary plaque assays was small (1.4–1.7x10^8^ PFU/mL), only a single technical replicate per biological replicate was performed. Of the 64 bacterial hosts tested, 6 biological replicates were obtained for 42 strains, 5 replicates for 12 strains, and 4 replicates for 10 strains.

### Assessing susceptibility using optical density assays

In each well of a 96-well plate, 180μL of LB Miller broth was added alongside 10μL of each *Staphylococcaceae* isolate diluted to an initial concentration of 1x10^6^ CFU/mL. For infected plates, 10μL of ISP at a concentration of 5x10^4^ PFU/mL was added to each well to achieve an MOI of 0.05 (final concentration of 5x10^4^ CFU/mL and 2.5x10^3^ PFU/mL). A low MOI was chosen to ensure any large changes in OD were due to multiple rounds of phage infection within the sample, requiring the production of viable phage progeny. For control plates, 10μL of 1X M9 buffer was added in place of ISP to maintain a final concentration of 5x10^4^ CFU/mL. Samples with and without phage were kept on separate plates to minimise the chance of phage contaminating uninfected samples. Within-plate position was kept constant between infected and control plates for each biological replicate but randomised between replicates to minimise the effect of within-plate position effects. The effects of between-plate variation were estimated by comparing OD readings between biological replicates of the same infection conditions (standard deviation of 0.14) and were found to account for a small amount of variation compared with the effect of infection (standard deviation of 0.67). Three LB Miller broth and three 1X M9 buffer controls were added to each plate to check for contamination. The plates were sealed with an adhesive PCR plate seal (Thermo Scientific) and incubated for 24 hours at 37°C, 180rpm. Following the incubation, OD was read at 600nm on a MultiSkan Sky Microplate Spectrophotometer (Thermo Fisher). To standardise for any differences in bacterial growth rate, susceptibility was calculated as a proportion change in OD due to infection, as follows:

$${OD}_{Change}=\frac{{OD}_{uninfected}-{OD}_{infected}}{{OD}_{uninfected}}$$

Several collected data points reported a proportion change in OD considerably less than zero. As this was likely the result of anomalous OD readings, outlier analysis was performed. Datapoints more than 1.5 times the interquartile range outside the upper and lower quartile of the data were considered minor outliers, and those more than 3 times the interquartile range outside the upper and lower quartiles were considered major outliers. All analyses included here were robust to the removal of both major and minor outliers (S2–S5 Tables), and so only data with all major and minor outliers removed is presented in the main text. Following outlier removal, 6 biological replicates were obtained for 56 of the bacterial strains tested, 5 replicates were obtained for 7 of the strains, and 3 replicates were obtained for 1 strain.

### Assessing susceptibility using quantitative PCR

Infections used to measure susceptibility using quantitative (q)PCR were set up identically to those in the OD assays described above. Again, a low MOI of 0.05 was used to ensure that any large changes in viral load were due to multiple rounds of phage infection, requiring the production of viable phage progeny. Following the 24 hour incubation, 100μL of each sample was transferred to a sterile 1.5mL screw cap microcentrifuge tube and heat treated at 90°C for 10-minutes to inactivate the bacteria. Additionally, 12 samples of the aliquot of ISP used to infect the *Staphylococcaceae* strains were diluted 1:20 in LB Miller broth and extracted to provide an initial viral load at infection timepoint zero. To extract the viral DNA, 100μL of 10% w/v Chelex 100 (Merck Life Sciences), 2ul of 20ng/μL Proteinase K (Merck Life Sciences), and ~10 1mm zirconia beads (Thistle Scientific) were added to each sample before mechanically lysing the bacteria using an Omni Bead Ruptor 24 (Camlab). Samples were centrifuged briefly, and heat treated for 10 minutes at 95°C to inactivate the Proteinase K before being centrifuged for a further 5 minutes at 18 x g to sediment out the Chelex. For each sample, 50μL of the supernatant containing bacterial and viral DNA was transferred and stored at -20°C.

qPCR was performed on each of the samples using an Applied Biosystems StepOnePlus system with a Sensifast Hi-Rox Sybr kit (Bioline). Cycle conditions were as follows: initial denaturation at 95°C for 120 seconds, then 40 cycles of 95°C for 5 seconds, and 60°C for 30 seconds. ISP was measured using the following primers: forward, 5’- CCTGTACCGGCTTGACTCTC -3’; reverse, 5’- AGCTACAACCGAGCAGTTAGA -3’, which were confirmed to have a near 100% efficiency when tested in the presence of each bacterial isolate. Pilot experiments showed that normalisation to either staphylococcal genomic DNA or an exogenous DNA spike made little difference to the between sample variation in ISP viral load and, therefore, no normalisation was used.

For each sample, two technical replicates of the qPCR reaction were performed. Amplification of the correct product was confirmed by melt curve analysis: samples that had failed to amplify the product, showed evidence of melt curve contaminants, or departed from the melt curve peak of positive samples by ±1.5°C were excluded. Correction for plate effects between technical replicates was performed using a linear model as previously described [91, 92]. Mean viral C_t_ values from technical replicate pairs (C_t:24_) were normalised to an initial dose of ISP (C_t:0_) and converted to fold change in viral load using the 2^–ΔCt^ method, where ΔCt = C_t:24_ –C_t:0_. Prior to analysis, these values were converted to a log_10_ fold change in viral load over 24 hours. Of the 64 bacterial strains tested, 6 biological replicates were obtained for 58 strains, 5 replicates for 5 strains, and 4 replicates for 1 strain.

### Inferring the host phylogeny

To infer the evolutionary relationships between *Staphylococcaceae* isolates, a core genome phylogeny was constructed using BEAST v1.10 [93]. Briefly (see S1 Text, S1–S4 Figs, and S6–S7 Tables for full details), whole genome sequences were collected for 8 previously sequenced strains from NCBI (S1 Table), and the remaining 56 sequences were obtained through whole genome sequencing. Library preparations and sequencing were performed by MicrobesNG (Birmingham, UK). Genomic DNA libraries were prepared using the Nextera XT Library Prep Kit (Illumina) with twice the stated amount of input DNA and PCR elongation increased to 45-seconds. Pooled libraries were quantified using the Kapa Biosystems Library Quantification Kit for Illumina and sequenced using Illumina sequencers (HiSeq/NovaSeq) with a 250-bp paired end protocol. Sequence reads were deposited on NCBI under the BioProject ID: PRJNA894984 (see S1 Table). Genomes were assembled and quality controlled as described in S1 Text.

D*e novo* assemblies were annotated using Prokka (v2.8.2) [94] and orthologous genes identified with Panaroo [95] using a sequence identity threshold of 0.7. Orthologous genes were then used to generate a core genome alignment of the 102 genes shared between each of the 64 *Staphylococcaceae* isolates. Phylogenetic trees were constructed using BEAST v1.10 [93]. Sequence alignments were fitted to a HKY substitution model using relaxed uncorrelated molecular clock models, gamma distributions of rate variation, and constant population size coalescent priors [93]. Separate substitution models and molecular clocks were fitted to 1^st^/2^nd^ and 3^rd^ codon positions, to reflect differences in selective constraint [96]. Two independent MCMC chains were run for each model until both convergence and a <10% burn-in was achieved. Convergence of all parameters was checked using Tracer v1.6 [97].

The within-*S*. *aureus* and among-species phylogenies were constructed by dropping the relevant tips from the 64-strain tree using the R package ape [98]. To ensure that the phylogenetic relationship between species and strains were accurately resolved in the 64-strain tree, separate phylogenetic trees were constructed using only the *S*. *aureus* samples or species isolates with 13S44S9 as a representative *S*. *aureus* strain (see S1 Text). As the topology of the smaller phylogenies matched that of the phylogenies made by dropping tips from the whole phylogeny, the dropped tip phylogenies were used for our analyses as they had comparable branch lengths to the 64-strain phylogeny.

### Phylogenetic mixed models

Phylogenetic generalised linear mixed models were used to investigate the effect of host relatedness on susceptibility to infection with ISP, and to examine correlations between the methods used to assess susceptibility. Multivariate models were fitted using the R package MCMCglmm [99] with susceptibility, as determined by each method (plaque assay, OD, and qPCR), as the response variable. Unscaled trees were used to correct for differences in the evolutionary divergence of our within- and among-species trees.

The structures of the model were as follows:

$$y_{him}=\beta_{1:m}+\mu_{p:hm}+\mu_{s:hm}+e_{him}$$ (1)

$$y_{him}=\beta_{1:m}+\mu_{p:hm}+e_{him}$$ (2)

In these models, *y*_*him*_ is the susceptibility measured by method *m* in the *i*^*th*^ biological replicate of host *h*. The fixed effect *β*_1_ represents the intercepts for each method, the random effect *μ*_p_ represents the effects of the host phylogeny assuming a Brownian model of evolution, and *e* represents the model residuals. Model (1) also includes a strain-specific random effect that is independent of the host phylogeny (*μ*_s:*hm*_). This explicitly estimates the non-phylogenetic component of between-strain variance and allows for the calculation of phylogenetic heritability (described below). *μ*_s:*hm*_ was removed from model (2) as model (1) failed to separate the phylogenetic and strain-specific effects. An additional version of model (1) was run with evolutionary distance from the propagation host added as a fixed effect (*β_d:hm_*). This was done to examine if the evolutionary distance from the propagation host explained any observed differences in susceptibility between strains, but was found to be non-significant and thus excluded from further models.

Susceptibilities measured by OD and qPCR were treated as normally distributed. However, to account for the zero-inflated nature of the plaque assay data, plaque assay susceptibility was divided into two separate variables–equivalent to a hurdle modelling approach–with a binary variable indicating whether the bacteria was permissive (1) or non-permissive (0) to infection, modelled using probit link function [100], and (conditional on being permissive) a continuous variable containing PFU/μL which was treated as normally distributed.

Within each of these models, the random effects and residuals were assumed to follow a multivariate normal distribution with a mean of 0 and covariance structure **V**_*p*_⊗**A** for the phylogenetic effects, **V**_*s*_⊗**I** for strain-specific effects, and **V**_*e*_⊗**I** for residuals, where ⊗ represents the Kronecker product. **A** represents the host phylogenetic relatedness matrix, **I** an identity matrix, and **V** represents 4 × 4 covariance matrices describing the variances and covariances in susceptibility for the different methods. Specifically, the matrices **V**_*p*_ and **V**_*s*_ describe the phylogenetic and non-phylogenetic between-strain variances in susceptibility for each method and the covariances between them, whereas the residual covariance matrix **V**_*e*_ describes the within-strain variance that includes both true within-strain effects and measurement errors. Here, between strain variation refers to the variation in susceptibility between bacterial strains, whereas within-strain variation refers to variation in susceptibility between biological replicates of the same strain (i.e., variation within the same strain), including both within-strain genetic variance and measurement error. Because each biological replicate consists of a measurement from a single method, the covariances of **V**_*e*_ cannot be estimated and were set to 0. Additionally, the residual variance for the binary variable cannot be estimated and was fixed at 1.

Models were run for 13 million MCMC generations, sampled every 5,000 iterations with a burn-in of 3 million generations. Parameter expanded priors were placed on the covariance matrices, resulting in multivariate F distributions with marginal variances being scaled by 1000. Inverse-gamma priors were placed on the residual variances, with a shape and scale equal to 0.002. To ensure the model outputs were robust to changes in prior distribution, models were also fitted with inverse-Wishart priors, which gave quantitatively similar results.

To estimate an effect of phylogeny on the susceptibility of *Staphylococcaceae* to ISP, we calculated several metrics. First, repeatability, used in the study of quantitative traits and defined as how ‘repeatable’ a measurement is within a group compared with measurements made across groups, was calculated from model (2) as **V**_*p*_/**V**_*p*_+**V**_*e*_, where **V**_*p*_ represents the phylogenetic (i.e., ‘across group’) variation, and **V**_*e*_ the within strain variation [101–103]. To estimate the proportion of between-strain variation that can be explained by phylogeny, we calculated phylogenetic heritability (*h*^2^) from model (1). Phylogenetic heritability is defined in two ways in the literature: either as the variance in *average* phenotype across strains that can be explained by phylogeny or as the variance in phenotype that can be explained by phylogeny [104, 105]. The former averages over the within-strain variation (**V**_*s*_) such that $h^{2}=\frac{V_{p}}{V_{p}+V_{s}}$ (i.e., the proportion of the phylogenetic and non-phylogenetic strain variance explained by phylogeny, referred to as “phylogenetic heritability” in this paper), whereas the latter does not and $h^{2}=\frac{V_{p}}{V_{p}+V_{s}+V_{e}}$ (i.e., the proportion of the *total* variance explained by phylogeny, defined as the “phylogenetic heritability of total variance” in this paper). Inter-strain correlations in viral load between each method were calculated from model (2) *V*_*p*_ matrix as $\frac{{cov}_{x,y}}{\sqrt{{var}_{x}\times {var}_{y}}}$ and the slopes (*β*) of each relationship as $\frac{{cov}_{x,y}}{{var}_{x}}$. Parameter estimates stated below are means of the posterior density, and 95% credible intervals (CIs) were taken to be the 95% highest posterior density intervals.

To visualise the outputs of our phylogenetic GLMMs as patterns of bacterial susceptibility across the host phylogeny, we plotted ancestral state reconstructions using methods described elsewhere (https://doi.org/10.6084/m9.figshare.7756526.v1). These plots take the trait values for each terminal node (i.e., the experimentally measured susceptibilities of extant bacterial hosts) and the inferred trait values of ancestral nodes from model (2) and plot them as colour gradients across the phylogeny, with changes in trait values along branches assumed to be smooth, fixed rate transitions as expected when following a Brownian model of trait evolution.

### Leave-one-out cross-validation

To investigate the ability of the bacterial host phylogeny to predict the susceptibility of a novel host, leave-one-out cross-validation was used [106], whereby multiple versions of model (1) were fitted, each with the data from a single bacterial strain removed, and the model challenged to predict the susceptibility of the “unknown” host given only its evolutionary relationships to other *Staphylococcaceae* strains and their measured susceptibilities. For comparison, a null model containing no effect of host phylogeny was fitted. Prediction errors from the leave-one-out cross-validation and the null model were compared using Wilcoxon rank sum tests [107] to determine whether information on the relationship between host species significantly improved the ability of the model to predict the susceptibility of an unknown host.

## Results

### ISP is a broad host range phage with varying infectivity across *Staphylococcaceae*

**Fig 1:**
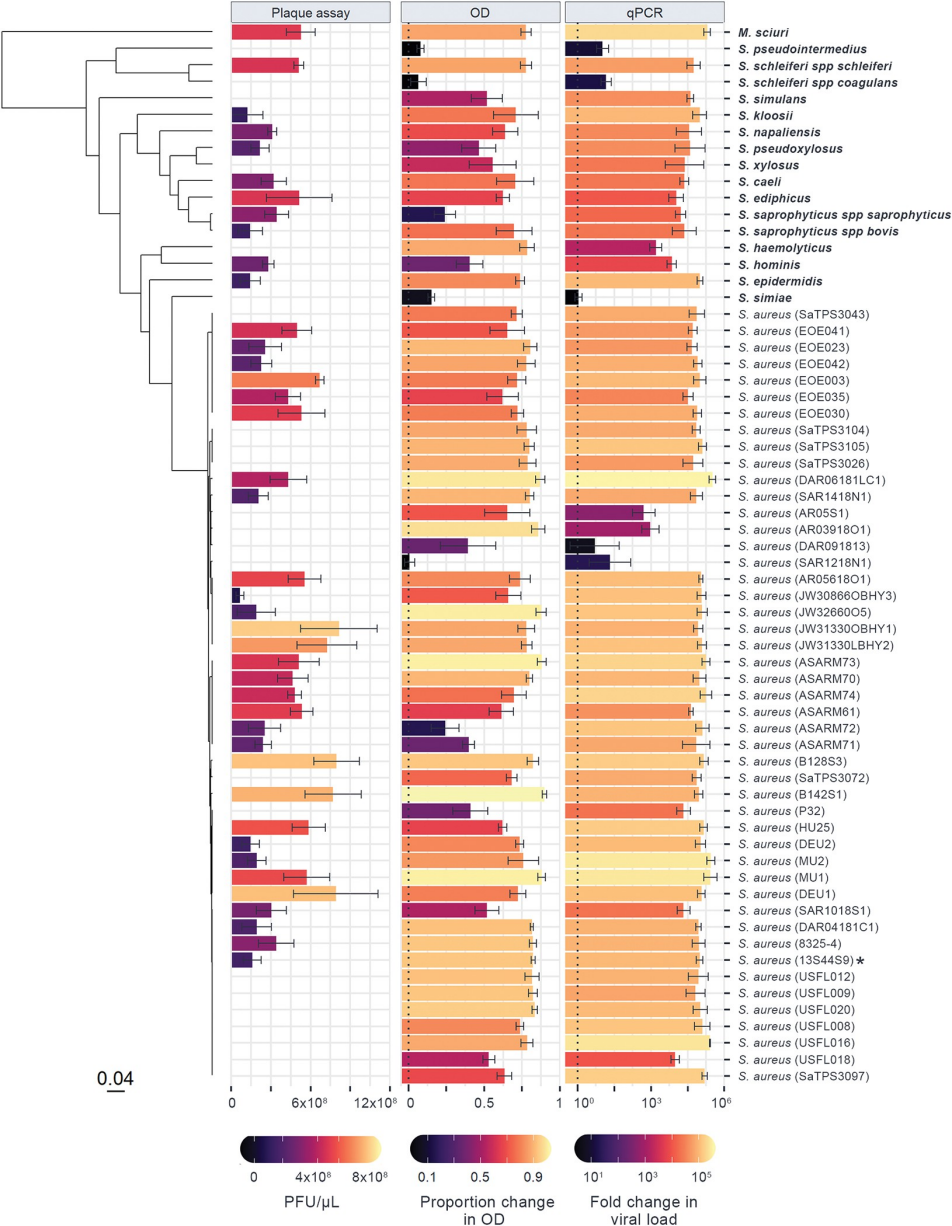
Susceptibility to ISP across a panel of 64 *Staphylococcaceae* isolates, measured using three distinct methods. Bar lengths and colour show the mean change in ISP assessed by plaque assay (PFU/μL), optical density (proportion decrease in OD with infection after 24 hours), and qPCR (log_10_ fold change in viral load after 24 hours), with error bars representing the standard error of the mean across at least four biological replicates. The phylogeny of *Staphylococcaceae* hosts is presented on the left, with the scale bar representing the number of nucleotide substitutions per site. Strain names are presented on the right, with non-aureus species in bold and the propagation host labelled with an asterisk (a full version of the tree is available at https://doi.org/10.6084/m9.figshare.21642209.v1).

To investigate the ability of the host phylogeny to explain variation in susceptibility to virus infection in bacteria–and how different methods may vary in their quantification of phage host range–we experimentally infected 64 strains of *Staphylococcaceae* with the bacteriophage ISP and assessed susceptibility using three distinct methods: plaque assays, OD assays, and qPCR. When assessed using plaque assays, 64% of host strains were seen to be permissive to infection with ISP. However, both OD and qPCR measures of susceptibility showed that ISP was capable of infecting 97% of the host panel (Fig 1). Variation in susceptibility between *Staphylococcaceae* isolates was seen in every method: the mean PFU/μL of permissive hosts ranged from 2.2x10^3^ in *S*. *simulans* to 8.1x10^5^ in *S*. *aureus* strain JW31330OBHY1; the mean proportional decrease in OD ranged from 0.01 in *S*. *aureus* strain SAR1218N1 to 0.90 in *S*. *aureus* strain B142S1; and the mean change in qPCR viral load ranged from a 1.2-fold increase in *S*. *simiae* to a ~300,000-fold increase in *S*. *aureus* strain DAR06181LC1 (Fig 1). Together, these results show that ISP is both able to infect a broad range of *Staphylococcaceae* strains and species, and that susceptibility to ISP varies widely across the *Staphylococcaceae* family.

### Susceptibility to ISP across *Staphylococcaceae* is explained by the host phylogeny

The phylogeny of *Staphylococcaceae* host species inferred here is broadly consistent with previous studies of these taxa [84], with the close phylogenetic relationships between species being generally well supported and hosts falling into two main clades (Fig 1). The *Staphylococcaceae* phylogeny is characterised by high susceptibility to ISP across a large number of isolates, with smaller clades showing reduced susceptibility to infection (i.e., the clade containing *S*. *aureus* strains AR05S1, AR03918O1, and DAR091813). Some clades show intermediate levels of susceptibility (e.g., the clade containing *S*. *haemolyticus* and S. *hominis*) while some contain both permissive and non-permissive strains (e.g., the clade containing *S*. *aureus* strains SAR1218N1 and AR05618O1).

**Fig 2:**
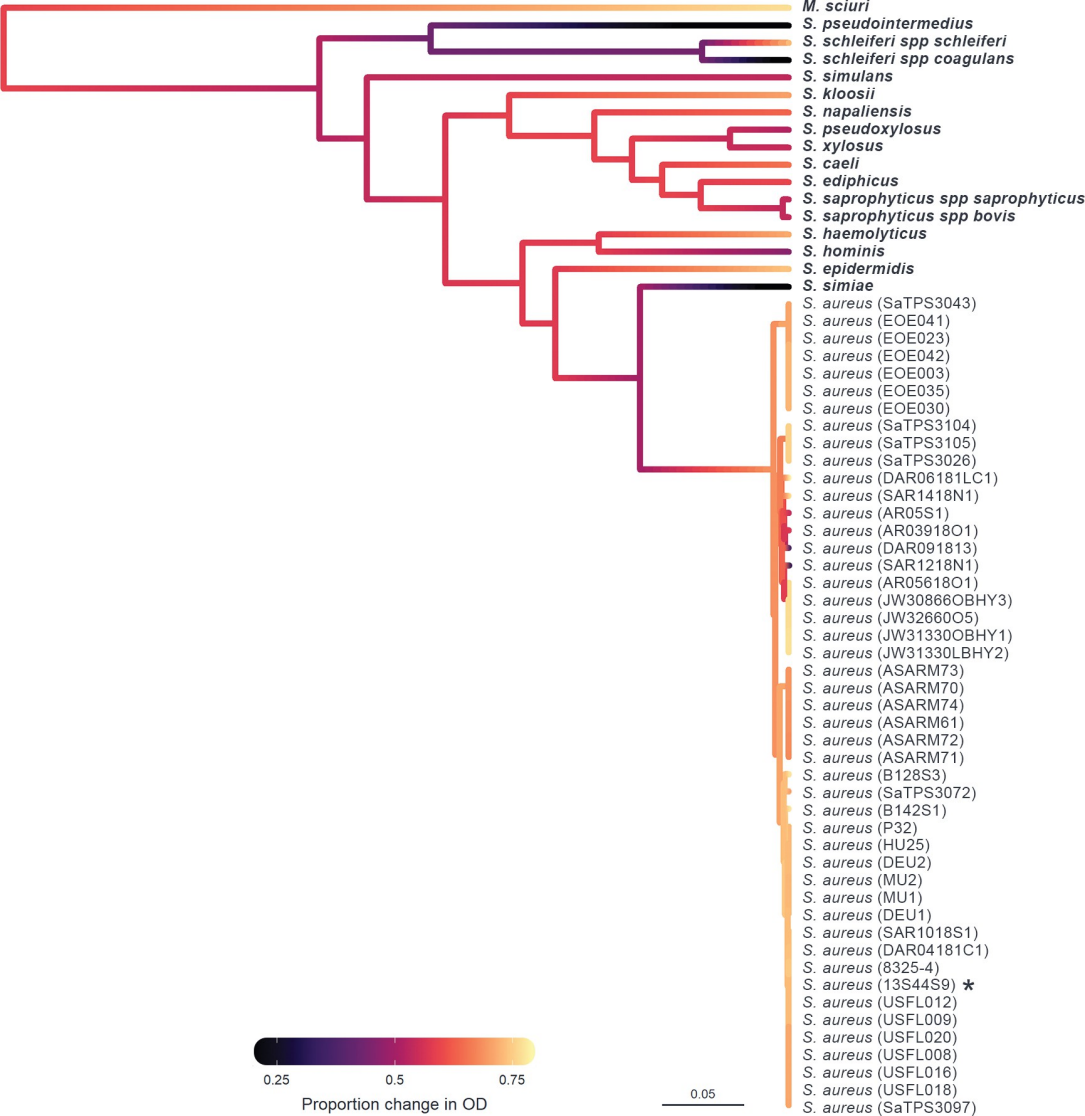
Ancestral state reconstruction of susceptibility to ISP measured by OD. Ancestral states were estimated from model (2) for each node and plotted in colour across the *Staphylococcaceae* host phylogeny, with the scale bar representing nucleotide substitutions per site. Colours represent susceptibility of a host to infection with ISP measured by OD (proportion change in optical density in infected compared to non-infected cultures), with black representing the lowest level of susceptibility and yellow the highest. Strain IDs are presented on the right, with non-aureus species in bold and the propagation host labelled with an asterisk (*).

**Fig 3:**
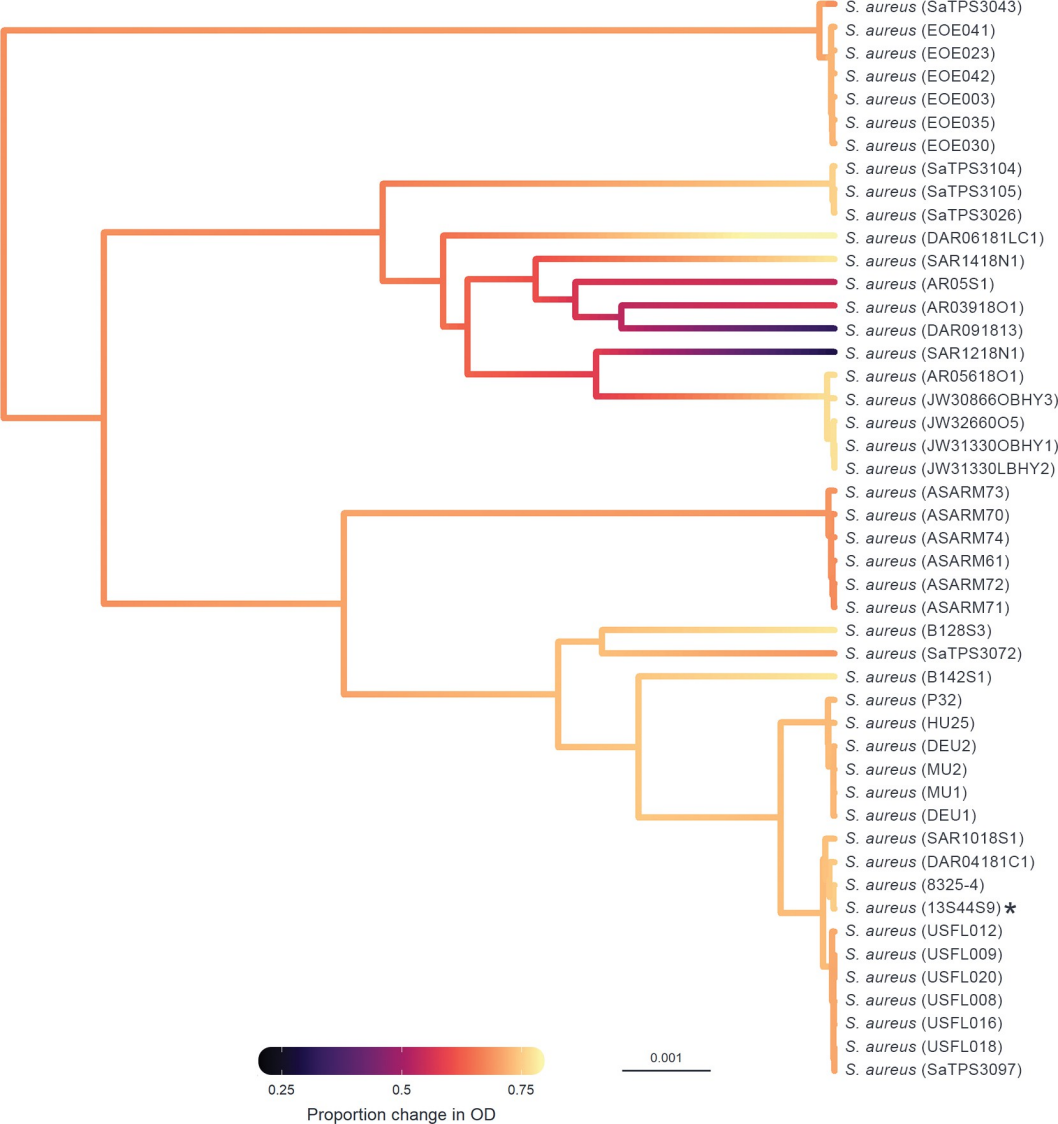
Ancestral state reconstruction of the *S*. *aureus* host strains susceptibility to ISP measured by OD. Ancestral states were estimated from model (2) for each node and plotted in colour across the *Staphylococcus aureus* host phylogeny, with the scale bar representing nucleotide substitutions per site. Colours represent susceptibility of a host to infection with ISP measured by OD (proportion change in optical density in infected compared to non-infected cultures), with black representing the lowest level of susceptibility and yellow the highest. Strain IDs are presented on the right and the propagation host labelled with an asterisk (*).

Phylogenetic generalised linear mixed models were fitted to the data to determine the proportion of variation in susceptibility explained by the host phylogeny (Table 1). Estimates of repeatability, phylogenetic heritability, and phylogenetic heritability of total variance for the binary plaque assay, OD, and qPCR data were close to 1 with narrow credible intervals. The convergence of phylogenetic heritability and repeatability estimates for the binary plaque assay, OD, and qPCR data at 1 suggests that the between-strain phylogenetic component explains a high proportion of the variation in susceptibility with little within-strain variation or measurement error (Table 1). Estimates of repeatability and phylogenetic heritability for the continuous (PFU/μL) component of the plaque assay had wide credible intervals spanning 0–1, suggesting that there is no effect of phylogeny when susceptibility is assessed using a continuous component of plaque assay. The effect of phylogeny on the susceptibility of *Staphylococcaceae* hosts to ISP can be further seen in ancestral state reconstructions (Figs 2 and 3), where different clades are seen to have similar susceptibilities to infection. This is particularly apparent within-*S*. *aureus* samples, where clades show either high, intermediate, or low susceptibility to ISP (Fig 3).

**Table 1:** Estimates for the repeatability and phylogenetic heritability across 64 *Staphylococcaceae* isolates. Estimates of repeatability are taken from model (2) and estimates of phylogenetic heritability (the proportion of phylogenetic and non-phylogenetic strain variation explained by the host phylogeny) and phylogenetic heritability of the total variance (the proportion of total variation explained by the host phylogeny) are taken from model (1). PA = plaque assay, CI = credible interval.

	Repeatability		Phylogenetic heritability		Phylogenetic heritability of total variance	
Method	Mean	95% CI	Mean	95% CI	Mean	95% CI
**Binary PA**	1.00	1.00, 1.00	1.00	1.00, 1.00	1.00	1.00, 1.00
**Continuous PA**	0.00	0.00, 0.00	0.68	0.01, 1.00	0.00	0.00, 0.00
**OD**	0.98	0.96, 0.99	0.97	0.94, 1.00	0.94	0.88, 0.98
**qPCR**	0.99	0.99, 1.00	1.00	1.00, 1.00	0.99	0.98, 1.00

To determine if the observed phylogenetic signal is consistent across evolutionary scales, we reduced the phylogeny to one containing only the *S*. *aureus* samples (S8 Table) and one containing each of the *Staphylococcaceae* species and a single representative *S*. *aureus* strain (13S44S9) (S9 Table). Similar estimates of repeatability were observed for both the within-*aureus* and among-species phylogeny models. However, both models showed a reduced ability to estimate phylogenetic effect, with wide credible intervals around estimates (apart from estimates for binary plaque assay and qPCR in the within-S. *aureus* model). It is likely that the observed difference in ability to estimate heritability between the whole phylogeny model compared to the reduced phylogeny models is down to reduced statistical power, causing the latter models to struggle to separate the phylogenetic and strain-specific effects.

Finally, to ensure that ISP had not adapted to its propagation host prior to the experiment, we looked for an effect of distance from the propagation host (*S*. *aureus* strain, 13S44S9). No effect of distance from the propagation host on the susceptibility of *Staphylococcaceae* to ISP was found (*β* = 0.03, 95% credible interval: -1.46, 1.56), suggesting that the observed phylogenetic signal was not being driven by adaptation of ISP to the propagation host.

### Measures of susceptibility from plaque assays, OD assays, and qPCR are positively correlated across hosts

Correlations between plaque assay, OD, and qPCR measures of susceptibility to ISP across bacterial host strains were estimated from the variance-covariance matrices of model (2) (Table 2). A strong positive inter-strain correlation was observed between susceptibility measured by OD and qPCR (*r* = 0.97, 95% CI: 0.92, 1:00), and a strong positive correlation was observed between the binary measure of plaque assay and OD (*r* = 0.94, 95% CI: 0.86, 1.00) and the binary measure of plaque assay and qPCR (*r* = 0.98, 95% CI: 0.94, 1.00). However, no evidence of a correlation was observed between the continuous plaque assay data and either OD or qPCR, with correlation coefficients approximately zero and credible intervals spanning -1 to 1 (Table 2). Similar correlations were observed between methods for the within *S*. *aureus* (S10 Table) and among-species (S11 Table) phylogenies, with the qPCR:OD correlation being the only one consistently and significantly positive. A correlation between the binary measure of PA and the other measures of susceptibility was observed in the among-species phylogeny but not the within-*S*. *aureus* phylogeny, suggesting that the correlation observed between these measures is being driven by strong correlations seen at the species level rather than the within-species level.

**Table 2:** Inter-strain correlations in susceptibility measures between methods. Numbers show the mean estimates for the correlation strength (*r*, white cells) and slope (*β*, grey cells) between pairs of methods, with 95% credible intervals (CIs) indicated in brackets. The slopes were calculated with the variables in columns as *x* and variables in rows as *y*. Estimates with CIs that do not span zero are highlighted in bold. PA = plaque assay, *value on a probit scale.

	Binary PA	Continuous PA	OD	qPCR
**Binary PA**	-	-	**0.94** **(0.86, 1.00)**	**0.98** **(0.94, 1.00)**
**Continuous PA**	-	-	-0.01 (-0.99, 0.99)	-0.01 (-1.00, 1.00)
**OD**	0.50* **(0.50, 0.50)**	-0.00 (-0.12, 0.17)	-	**0.97** **(0.92, 1.00)**
**qPCR**	0.51* **(0.50, 0.52)**	0.01 (-1.30, 0.97)	**7.45** **(5.53, 9.59)**	-

### Mixed evidence for host phylogeny improving the accuracy of susceptibility predictions

**Fig 4:**
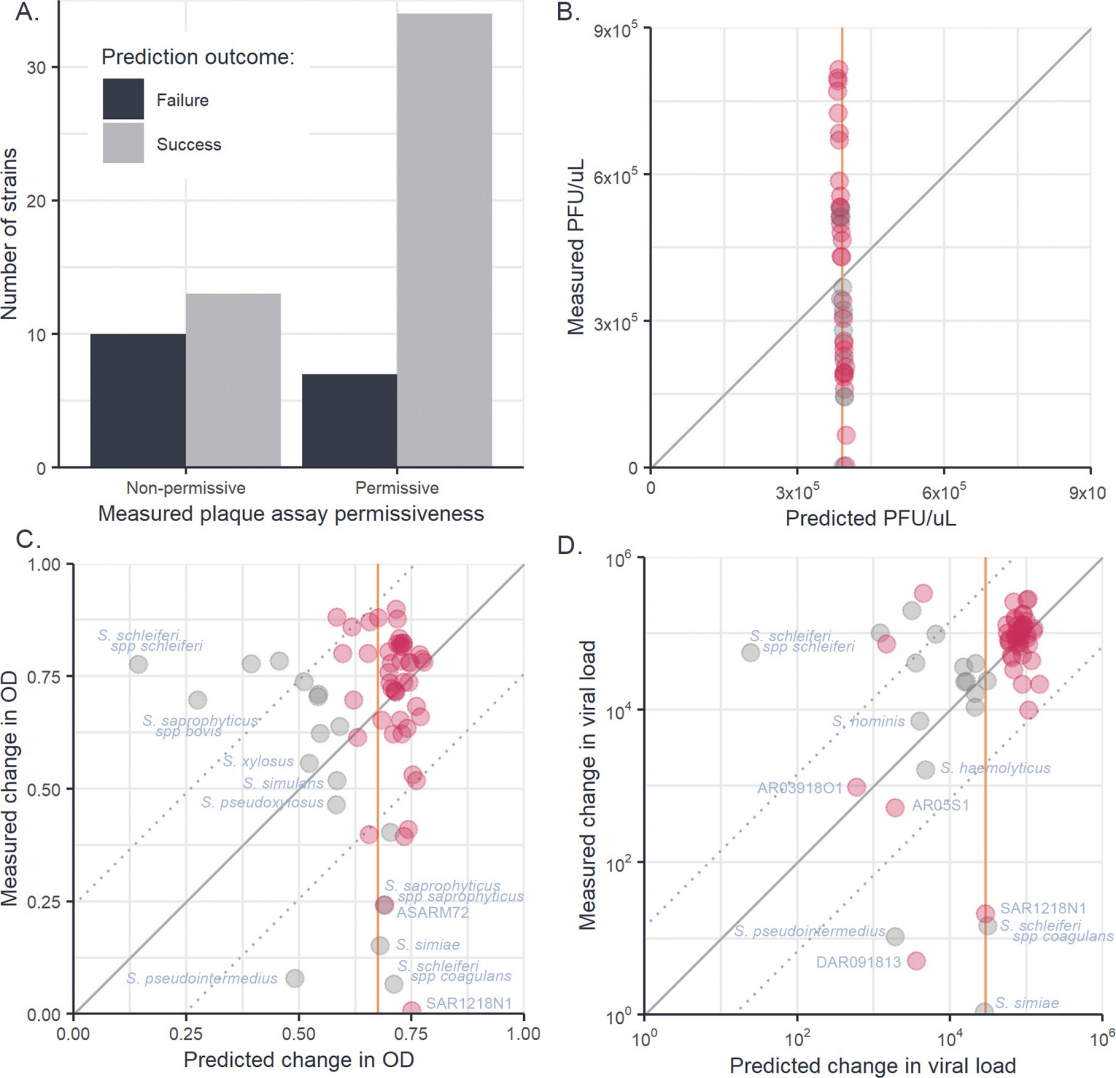
Leave-one-out cross-validation of phylogenetic mixed models fitted to plaque assay, OD, and qPCR data. Predictions of “unknown” trait values from a leave-one-out cross-validation of phylogenetic model (2) for binary plaque assay (A), continuous (PFU/μL) plaque assay (B), change in OD with infection (C) and fold changes in viral load measured by qPCR (D). Each datapoint represents an individual strain of *Staphylococcaceae* whose measured trait value has been removed from model (2) and predicted from its evolutionary relationships to other *Staphylococcaceae* isolates and their trait values. Solid diagonal lines illustrate the location of 1:1 predictions and dotted lines indicate the root-mean-squared errors around these lines. Orange vertical lines represent the predicted trait values of all strains from a null (intercept only) model. For panels B, C, and D, points plotted in pink show the *S*. *aureus* strains whereas points plotted in grey show the non-*S*. *aureus* strains.

As the host phylogeny explains a large proportion of the variation in ISP susceptibility, it may allow for the susceptibility of untested *Staphylococcaceae* strains and species to be predicted based on their evolutionary relationships to staphylococci with known susceptibilities. To investigate the ability of the bacterial host phylogeny to predict susceptibility, leave-one-out cross-validation was used, whereby multiple versions of model (2) were fitted, each with the data from a single bacterial strain removed, and the model challenged to predict the susceptibility of the “unknown” host given only its evolutionary relationships to other staphylococci strains and their measured susceptibilities (Fig 4).

For the binary component of plaque assay, the phylogenetic model was able to correctly predict a strain’s permissiveness to ISP infection in 73% of cases (Fig 4A), whereas a null model without phylogeny predicted all hosts to be permissive, achieving an accuracy of 63%. When asked to predict the continuous (PFU/μL) component of plaque assays, errors from the phylogenetic and null models were indistinguishable (Wilcoxon rank sum test: W_43,43_ = 886, p = 0.74), as in the phylogenetic model most of the variation in PFU/μL was partitioned into the model residuals, causing trait values predicted from the phylogenetic component to cluster around the across-strain mean (Fig 4B). When asked to predict susceptibility measured by OD assay (Fig 4C), the errors produced by the phylogenetic model were also not significantly different from those produced by a null model (Wilcoxon rank sum test: W_64,64_ = 2111, p = 0.77). However, when predicting viral loads from qPCR data (Fig 4D), the phylogenetic model produced a small but significant decrease in error compared to the null model (Wilcoxon rank sum test: W_64,64_ = 2726, p < 0.01).

In the OD and qPCR assays most strains were both similar in susceptibility to their close relatives and showed susceptibility close to the across-strain mean (Figs 1, 4C, and 4D), causing the phylogenetic and null models to produce similar predictions for the majority of strains. In cases where hosts existed in clades with susceptibilities different from the across-strain mean, prediction accuracy increased when phylogeny was included (e.g., qPCR prediction for *S*. *haemolyticus* and *S*. *hominis*, OD prediction for *S*. *xylosus* and *S*. *pseudoxylosus*). However, when hosts had susceptibility near the across-strain mean and had close relatives that differed from this mean, the phylogenetic model produced larger errors in prediction than the null model (e.g., both OD and qPCR predictions for *S*. *schleiferi spp schleiferi*). Lastly, where hosts differed from the across-strain mean but had close relatives that conformed to the mean, both phylogenetic and null models were poor predictors of susceptibility (e.g., both OD and qPCR predictions for *S*. *schleiferi spp coagulans* and *S*. *aureus* SAR1218N1). Together, these results suggest that the host phylogeny may allow for the limited prediction of host susceptibility, but may mislead predictions in host strains that differ strongly from the susceptibilities of their close relatives.

## Discussion

Closely related host species present similar environments to novel viruses [103, 108], and so tend to share similar levels of susceptibility [17, 19–21, 26]. Here, we have examined how susceptibility to a broad host range phage varies across a diverse panel of *Staphylococcaceae* bacteria and determined what proportion of the variation in phage susceptibility is explained by the relationships between bacterial hosts. ISP was capable of infecting 97% of the host strains investigated here when measured by OD and qPCR assays, but only 64% of these strains appeared permissive when tested by plaque assay. We found that variation in susceptibility across our *Staphylococcaceae* panel–measured using OD, qPCR, and the binary component of plaque assays–was largely explained by the host phylogeny, and these effects were seen at both within-species, and among-species phylogenetic scales. No effects of evolutionary distance from the propagation host were seen, with the patterns instead being driven by the existence of host clades sharing similar levels of susceptibility to ISP. Strong positive correlations were seen between the susceptibility measures from the binary plaque assay, OD, and qPCR assays, but not between these methods and the PFU/μL measures taken from plaque assays. Together, these results suggest that ISP has a broad ability to infect bacterial hosts within the *Staphylococcaceae* family, and that a large proportion of the variation in susceptibility to ISP across *Staphylococcaceae* hosts can be explained by the evolutionary relationship between bacterial hosts.

Recent studies investigating the host range of phages have employed host panels of varying phylogenetic scales, including analyses across diverse panels of bacterial species [51, 53, 57, 58, 109–111], and focused investigations in a high number of isolates within a single species [54, 55, 59]. This variable diversity of hosts selected for host range screening has often affected our interpretation of host range, with “broad host range” used to refer to both phages able to infect many strains within a single species and phage able to infect a large number of species within a genus. Here, we have demonstrated that ISP is capable of infecting two genera and 17 species within the S*taphylococcaceae* family. This expands upon the findings of a previous study which demonstrated that ISP was able to infect many strains of *S*. *aureus* [67] and extends the number of hosts that the phage can infect. The identification of phages with a broad host range is advantageous for phage therapy, where broad range phage can be used to treat a larger number of clinical infections. Having such phages available for study would also be beneficial to our growing understanding of virus host shifts, as it has been demonstrated that viruses with a broad host range are more likely to shift between species [112].

Both permissiveness and susceptibility of bacterial hosts to phage are key considerations when designing phage therapies. In our results, variation in permissiveness and susceptibility to ISP was apparent, both within *S*. *aureus* and among *Staphylococcaceae* species. This among-strain and among-species variation is likely due to differences in the molecular pathways influencing host range. For example, previous studies on phages have demonstrated that the highly conserved wall teichoic acid (WTA) serves as the primary receptor for staphylococcal phages [79], contributing to their broad host range. Further studies have characterised the anti-phage defence systems present in *S*. *aureus* and found that a combination of type I and type III restriction modification systems prevent the uptake of DNA originating from other species [113] and between *S*. *aureus* lineages [114], respectively, meaning that patterns of restriction can be clonal complex specific and lead to independent lineage evolution [115]. A Genome-wide association study (GWAS) aiming to identify genes associated with susceptibility measured by plaque assays identified putative loci affecting the host range of *S*. *aureus* bacteriophages, including: the TarJ, TagH, and TarP proteins which are involved in the production and transport of WTA to the bacterial cell surface; HsdS which determines the sequence specificity of the SauI restriction modification system; and several prophage associated genes, suggesting that superinfection immunity is playing a role in the susceptibility of *S*. *aureus* to bacteriophage [63]. Given the increase in phage host range observed in our study when susceptibility was assessed by either OD or qPCR, it is likely that future GWAS approaches based on susceptibility assessed by different methods may reveal further molecular pathways involved in bacteriophage host range. Further investigation of ISP to identify the specific molecular pathways by which it interacts with its host will improve our understanding of the factors that contribute to variation in susceptibility across bacterial hosts, allowing us to utilise ISP more efficiently as a therapeutic.

Bacterial evolution is known to involve several processes that could disrupt the phylogenetic heritability of susceptibility to phage. Notably, cell surface receptors can be easily altered by point mutations and components of phage resistance may be frequently gained and lost through horizontal gene transfer, which can lead to high levels of variation in the phage defence systems present in bacterial genomes [33, 116–120]. Previous investigations have shown that the carriage of prophage and the transfer of restriction modification systems can be lineage specific in bacteria [36, 115]. However, the extent to which these mechanisms are phylogenetically constrained is unknown. Despite the potential for horizontal gene transfer and large effect point mutations to disrupt the phylogenetic signal in phage susceptibility, our models suggest that the evolutionary relationships between a diverse panel of bacterial hosts can capture a large proportion of the variation in susceptibility to ISP. If horizontal gene transfer is occurring more frequently between closely related strains of *Staphylococcaceae*, then their influence on phage susceptibility may be phylogenetically conserved and this variation may have been captured in the core genome phylogeny. In any case, our results indicate that more closely related *Staphylococcaceae* strains and species are more likely to share similar susceptibilities to phage infection, consistent with patterns seen in previous studies of animal hosts and viruses [17, 19–21, 26]. Additionally, while the evolutionary relationship between hosts had been shown to influence susceptibility within a single species of *Staphylococcus* [63], we have found that the relationship between hosts can explain susceptibility over a much broader phylogenetic scale, across genera. Further work should aim to characterise the mechanisms underpinning the differences in susceptibility reported here. In particular, the role of horizontal gene transfer [33], superinfection immunity [35], and recombination [34] in the resistance of *Staphylococcaceae* to phage infection, as well as the evolutionary scale at which the relationship between host species is unable to explain variation to susceptibility.

In this study, we used three methods to assess the susceptibility of *Staphylococcaceae* to a bacteriophage: plaque assays, optical density assays, and qPCR. Susceptibility measured by binary plaque assay, OD, and qPCR were strongly positively correlated. However, no correlation was seen between the continuous component of plaque assay and either other method. While OD and qPCR suggest a broad host range for ISP, plaque assays underestimated the susceptibility of the host panel, with only 63% of strains appearing permissive to infection. This discrepancy may in part be due to differences in the environmental conditions imposed by each assay on the bacteria and phage. Bacteria in the plaque assay are sessile and aerobic, whereas bacteria are planktonic and anaerobic in the OD and qPCR assays. For facultative anaerobes, such as *Staphylococcus*, anoxia has been shown to lead to the differential expression of over 200 genes influencing a multitude of biological processes [121–126]. While the ability of phage to infect *Staphylococcaceae* species under varying oxygen concentrations is understudied, it is likely that differences in oxygen availability would influence bacterial physiology in a way that may affect phage infection [125, 127]. Further investigation into the influence of oxygen availability on phage infection, and the physiological relevance of this to various clinical infections may improve our ability to effectively utilise ISP as a therapeutic and explain the lack of correlation between the plaque assays and OD and qPCR used here. Interestingly, we observe a strong positive correlation between OD and qPCR even though both assays are measuring different aspects of infection, with OD providing a measure of damage done to the host and qPCR a measure of virus replication and persistence. That these two infection traits are strongly correlated suggests that staphylococcal phage immunity is strongly tied to their ability to disrupt and prevent phage replication, as opposed to mitigating damage while tolerating phage replication.

Being able to predict and prevent emerging infectious diseases is a major goal of scientific research, and one of the first steps of that process is being able to predict whether a host will be susceptible to a novel pathogen [128]. While our phylogenetic models suggest that a high proportion of the variation in susceptibility between host species can be attributed to the relationship between hosts, they showed a limited ability to predict the susceptibility of an unknown host given only its relationship to other hosts and their susceptibilities. For example, *S*. *simiae* is isolated within the phylogeny, with few close relatives, and had a poorly estimated susceptibility due to the distance between it and its nearest neighbour. Several additions may improve the accuracy of predictions of susceptibility using these models. Firstly, improving the depth of our phylogeny–i.e., adding more strains and species that reduce the number of isolated strains on long evolutionary branches–would increase the number of observations that the model can use to predict susceptibility. Secondly, furthering our understanding of the mechanisms by which susceptibility can change in a non-phylogenetically tractable way (i.e., via HGT, superinfection immunity, or recombination) may improve our ability to predict susceptibility when phylogeny is not informative. Predictive models that incorporate the evolutionary relationships between hosts (core genome phylogeny) alongside additional information about the presence of anti-phage defence systems and prophages may be better able to predict susceptibility in instances where phylogeny alone is not informative. Alternative metrics, such as genome composition bias, have been shown to outperform phylogeny in the prediction of infection traits [129], and may make useful additions to future predictive models [128–130]. Understanding the complexity of factors that contribute to viral susceptibility, and how they interact with one another, is an important step towards the prediction of susceptibility in unknown hosts. An ability to predict the susceptibility of a host to novel pathogens, particularly *how* susceptible a host will be to infection, would allow us to better prioritise resources to the prevention and control of emerging infectious diseases in humans and wildlife.

Together, our results demonstrate that the bacterial host phylogeny is an important determinant of phage susceptibility and replication across novel hosts, and that the relationship between host species may be a useful addition to models aiming to predict virus host range both in the context of emerging infectious disease and phage therapy. Further work is required to understand the specific interactions underlying variation in ISP susceptibility across bacterial hosts; the relationship between host damage, virus replication, and virus persistence in this system; and how the patterns of phage susceptibility across bacterial phylogenies may vary under different infection conditions and contexts.

## Supporting information

S1 Text Supplementary methods. (DOCX)

S1 Table *Staphylococcaceae* metadata and sequencing data. Clonal complex and sequence type, where available, were determined using PubMLST [23]. *S. aureus* strains missing a ST are coagulase negative and therefore do not have an MLST scheme and the *S. aureus* strains with STs but no CCs are not similar enough to other strains to be assigned a CC.
(DOCX)

S2 Table Estimates for the repeatability and heritability across 64 *Staphylococcaceae* isolates when no outliers were removed from the OD data. Estimates of repeatability are taken from model (2) and estimates of phylogenetic heritability (the proportion of phylogenetic and non-phylogenetic strain variation explained by the host phylogeny) and phylogenetic heritability of the total variance (the proportion of total variation explained by the host phylogeny) are taken from model (1). PA = plaque assay, CI = credible interval.
(DOCX)

S3 Table Estimates for the repeatability and heritability across 64 *Staphylococcaceae* isolates when major outliers were removed from the OD data. Estimates of repeatability are taken from model (2) and estimates of phylogenetic heritability (the proportion of phylogenetic and non-phylogenetic strain variation explained by the host phylogeny) and phylogenetic heritability of the total variance (the proportion of total variation explained by the host phylogeny) are taken from model (1). PA = plaque assay, CI = credible interval.
(DOCX)

S4 Table Estimates from the phylogenetic generalised linear mixed models for inter-strain correlations in susceptibility between methods where no outliers were removed from the OD data. Numbers show the mean estimates for the correlation strength (r, white cells) and slope (β, grey cells) between pairs of methods, with 95% credible intervals (CIs) indicated in brackets. The slopes were calculated with columns as x and rows as y. Estimates with CIs that do not span zero are highlighted in bold. PA = plaque assay, *value on a probit scale.
(DOCX)

S5 Table Estimates from the phylogenetic generalised linear mixed models for inter-strain correlations in susceptibility between methods where major outliers were removed from the OD data. Numbers show the mean estimates for the correlation strength (r, white cells) and slope (β, grey cells) between pairs of methods, with 95% credible intervals (CIs) indicated in brackets. The slopes were calculated with columns as x and rows as y. Estimates with CIs that do not span zero are highlighted in bold. PA = plaque assay, *value on a probit scale.
(DOCX)

S6 Table *Staphylococcaceae* assembly QC. (DOCX)

S7 Table *Staphylococcaceae* sequences used in the FastQ Screen to determine if all sequences used in this study belong to *Staphylococcaceae*. (DOCX)

S8 Table Estimates from the *S. aureus* only model for the repeatability and heritability. Estimates of repeatability are taken from model (2) and estimates of phylogenetic heritability (the proportion of phylogenetic and non-phylogenetic strain variation explained by the host phylogeny) and phylogenetic heritability of the total variance (the proportion of total variation explained by the host phylogeny) are taken from model (1). PA = plaque assay, CI = credible interval.
(DOCX)

S9 Table Estimates from the species only model for repeatability and heritability. Estimates of repeatability are taken from model (2) and estimates of phylogenetic heritability (the proportion of phylogenetic and non-phylogenetic strain variation explained by the host phylogeny) and phylogenetic heritability of the total variance (the proportion of total variation explained by the host phylogeny) are taken from model (1). PA = plaque assay, CI = credible interval.
(DOCX)

S10 Table Inter-strain correlations between methods for assessing host range in a within-*S. aureus* model. Numbers show the mean estimates for the correlation strength (r, white cells) and slope (β, grey cells) between pairs of methods, with 95% credible intervals (CIs) indicated in brackets. The slopes were calculated with columns as x and rows as y. Estimates with CIs that do not span zero are highlighted in bold. PA = plaque assay, *value on a probit scale.
(DOCX)

S11 Table Inter-species correlations between methods for assessing host range in an among-species model. Numbers show the mean estimates for the correlation strength (r, white cells) and slope (β, grey cells) between pairs of methods, with 95% credible intervals (CIs) indicated in brackets. The slopes were calculated with columns as x and rows as y. Estimates with CIs that do not span zero are highlighted in bold. PA = plaque assay, *value on a probit scale.
(DOCX)

S1 Fig A comparison of all 123 trees on 64 tips using the Kendall Colijn metric vector (mid-point rooted gene trees). MDS visualization of tree distances when λ  =  0. As the projection often requires that multiple trees are plotted at the same co-ordinates, contour lines are used to indicate the density of points.
(DOCX)

S2 Fig Core genome phylogeny of 64 *Staphylococcaceae* samples with the posterior probabilities of the MCMC chain displayed. (DOCX)

S3 Fig A subset of the core genome phylogeny showing 17 non-*S. aureus Staphylococcaceae* samples with the posterior probabilities of the MCMC chain displayed. (DOCX)

S4 Fig A subset of the core genome phylogeny showing 47 *S. aureus* samples with the posterior probabilities of the MCMC chain displayed. (DOCX)
