# Supplementary material for: The host phylogeny determines viral infectivity and replication across Staphylococcus host species
Source: PLoS Pathog. 2023 Jun 8;19(6):e1011433. doi: 10.1371/journal.ppat.1011433 (PMC10284401; doi:10.1371/journal.ppat.1011433)
Supplement: S1 Text — (DOCX) [file ppat.1011433.s001.docx]

**S1 Text**

## Supplementary Methods

## **Sample preparation and DNA extraction**

To infer the relationship between host species, 56 of the *Staphylococcaceae* isolates were whole genome sequenced. Pure cultures of each of the strains were grown on LB agar plates at 37^o^C overnight. Bacteria were then harvested from the plates and resuspended in a 2mL tube containing cryoperservative (Microbank, Pro-Lab Diagnostics UK) according to MicrobesNG instructions. Samples were then shipped to MicrobesNG where they were processed as follows: 5-40uL of the bacterial suspension were lysed with 120uL of TE buffer containing lysozyme (final concentration 0.1mg/mL) and RNase A (ITW Reagents) (final concentration 0.1mg/mL) and incubated for 25 minutes at 37^o^C. Proteinase K (VWR Chemicals) (final concentration 0.1mg/mL) and SDS (Sigma-Aldrich) (final concentration 0.5% v/v) are added and incubated for 5 minutes at 65^o^C. Genomic DNA was then purified using an equal volume of SPRI beads and resuspended in EB buffer (Qiagen). DNA was quantified using a Quant-iT dsDNA HS kit (ThermoFisher Scientific) assay in an Eppendorf AF2200 plate reader (Eppendorf UK Ltd).

1. **Illumina sequencing**

Library preparation and sequencing were performed by MicrobesNG (Birmingham, UK). Briefly, genomic DNA libraries were prepared using the Nextera XT Library Prep Kit (Illumina) with the following modifications: twice the amount of input DNA is used, and PCR elongation is increased to 45-seconds. Pooled libraries were quantified using the Kapa Biosystems Library Quantification Kit for Illumina and sequenced using Illumina sequencers (HiSeq/NovaSeq) with a 25-bp paired end protocol.

1. **Whole genome sequence quality control and *de novo* assembly**

To supplement the 56 samples that were sequenced by MicrobesNG, the whole genome sequences of the remaining 8 samples were downloaded from NCBI (S1 Table). Adapters and low-quality reads were removed with *Cutadapt* v1.16 (1) and *Sickle* v1.33 (2) and reads screened for contamination using FastQ Screen v0.12.0 (3). *De novo* assembly was performed using the SPAdes genome assembler (v3.9.1) (4). Optimal k-mers were identified based on the average read lengths for each genome.

All assemblies were evaluated using QUAST v.5.0.2 (5) and reads mapped back to *de novo* assemblies to identify potential contamination using *Bowtie2* v2.3.4.1 (6). Low-quality genome assemblies were excluded from further analysis, defined in the following way: all assemblies with an N50 of less than 10kb; *S. aureus* total sequence length plus or minus one standard deviation outside of the median sequence length based on previously collected data of 2011 *S. aureus* isolates (the median and standard deviation of genome size for other *Staphylococcaceae* species was evaluated based on published genomes); all assemblies with greater than 1500 SNPs (this threshold was defined by identifying a distribution of SNPs in all assemblies); and finally, if the total length of contigs smaller than 1kb exceeded 15% of the total genome assembly length (often indicative of a sample with >1 bacterial isolate), but only if one or more other QC measure was not met. Assembly QC information is presented in S6 Table.

A FastQ Screen v0.12.0 was used to check post-sickle reads for contamination and to determine the identification of a *Staphylococcus* species. This was further confirmed via Kraken (7,8) by creating a custom database of *Staphylococcus* reference genomes (S7 Table) along with a built-in database with PhiX of vectors and contaminants commonly seen in sequencing experiments (8). The custom database was created by downloading the *Staphylococcus* reference genome sequences in FASTA format from NCBI and then using Bowtie2 to build the relevant index files (9). Contaminants were detected by comparing the percentage of reads mapped uniquely against each of the reference genomes and additionally investigated using Kraken. Contamination was assumed if the reads mapped to a reference strain <70% and mapped to other species >30%. Ten isolates were identified in FastQ screen below >70% match and nine of these were <70% in Kraken. 271Y was mapped to *Staphylococcus kloosii* at 67.38% but this specific species only has 18 genomes available on NCBI, which most likely does not represent the species diversity and as other QC measures were within acceptable range was not excluded.

1. **Construction of a phylogenetic tree for the 48 *S. aureus* strains**

For the phylogenetic tree containing only the *S. aureus* isolates, reference-mapped assemblies were generated using Bowtie2 v1.2.2. In order to identify a reference strain outside of the collection diversity, sequence types (STs) for each *S. aureus* genome were identified using MLST-check (10). The reference genome S0385 was selected as none of the *S. aureus* were identified as ST398 (GenBank accession no. AM990992) (9,11). In order to map completed reference genomes, artificial FASTQ files were generated using ArtificialFastqGenerator (12).

Recombination was identified in the reference-mapped alignment using both Gubbins v2.3.1 (13) and ClonalFrame (14). All identified recombinant sites were masked, including a previously identified ∼123 kb genome segment horizontally acquired from an ST9 donor (15).

Phylogenetic reconstruction was carried out for the reference-mapped alignment with RAxML (v8.2.4) using the GTR+Γ model and 1,000 bootstraps (16) with a *Staphylococcus argenteus* outgroup (MSHR1132) to establish the root. The phylogenetic reconstruction was then carried out excluding the outgroup. To only include phylogenetically informative sites, sites containing more than a single gap were excluded due to marked recombination or missing data. Trees were visualized and annotated with Figtree (http://tree.bio.ed.ac.uk/software/figtree/) and the Interactive Tree Of Life (17).

1. **Construction of a core genome alignment for the *Staphylococcaceae*** **host panel**

D*e novo* assemblies were annotated using Prokka (v2.8.2) (18) and orthologous genes identified with Panaroo (19) using a range of different sequence identity thresholds (0.95- 0.70). A sequence identity threshold of 0.7 was selected to maximise the number of orthologous groups with only one representative in each of the 64 isolates, while reducing the homology groups containing duplicates. This threshold identified 129 genes, of which six were excluded due non-orthologous groups or having at least a single gap within all the sites in the gene alignment. Each of the remaining 123 identified orthologous groups was investigated by creating a single gene neighbour joining tree using the ape package in R and a K80 substitution model (20). Contradictory gene topologies were identified using the R package “treescape” (21) as previously described by (22) using a multi-dimensional scaling visualization of tree distances (S1 Fig). From this, 21 of the genes with the most district tree topologies were excluded. The final core genome alignment was created based on 102 genes (125,893 bp).

1. **Construction of an ultrametric phylogenetic tree for the *Staphylococcaceae*** **host panel**

An ultrametric phylogenetic tree was constructed using BEAST v1.10 with an HKY+Γ model, an uncorrelated relaxed molecular clock, and constant population size coalescent prior, from the reference-mapped alignment for *S. aureus* and core genome for all *Staphylococc*i spp (23). For the *S. aureus* reference-mapped phylogenetic reconstruction we fitted separate substitution models and molecular clocks to 1st/2nd, and 3rd codon positions, non-coding and RNA positions to reflect differences in selective constraint (23). For the core genome Staphylococci phylogenetic reconstruction, we fitted separate substitution models and molecular clocks to 1st/2nd, and 3rd codon positions, to reflect differences in selective constraint (23). For the two models, we ran two independent MCMC until convergence was reached and the burn-in represented <10% of the chain. Convergence of all parameters was checked in the program Tracer v1.4 (24). Posterior probabilities of the MCMC chain can be seen in S2-S4 Figs.

Supplementary references

1. Martin M. Cutadapt removes adapter sequences from high-throughput sequencing reads. EMBnet.journal. 2011 May;17(1):10.

2. Joshi N, Fass J. Sickle: A sliding-window, adaptive, quality-based trimming tool for FastQ files (Version 1.33) [Software]. Available at https://github.com/najoshi/sickle. 2011;2011.

3. Wingett S. FastQ Screen - Contamination screening for NGS data. http://www.Bioinformatics.Babraham.Ac.Uk/Projects/fastq-screen/. 2011.

4. Bankevich A, Nurk S, Antipov D, Gurevich AA, Dvorkin M, Kulikov AS, et al. SPAdes: a new genome assembly algorithm and its applications to single-cell  sequencing. Journal of computational biology : a journal of computational molecular cell biology. 2012 May;19(5):455–77.

5. Gurevich A, Saveliev V, Vyahhi N, Tesler G. QUAST: Quality assessment tool for genome assemblies. Bioinformatics. 2013 Apr;29(8):1072–5.

6. Langmead B, Salzberg SL. Fast gapped-read alignment with Bowtie 2. Nature Methods. 2012 Mar;9(4):357–9.

7. Wood DE, Salzberg SL. Kraken: Ultrafast metagenomic sequence classification using exact alignments. Genome Biology. 2014 Mar;15(3):1–12.

8. Wingett S. FastQ Screen - Contamination screening for NGS data. http://www.Bioinformatics.Babraham.Ac.Uk/Projects/fastq-screen/. 2011.

9. Langmead B, Salzberg SL. Fast gapped-read alignment with Bowtie 2. Nature Methods. 2012 Mar;9(4):357–9.

10. Page AJ, Taylor B, Keane JA. Multilocus sequence typing by blast from de novo assemblies against PubMLST. The Journal of Open Source Software. 2016;1(8).

11. Schijffelen MJ, Boel CE, van Strijp JA, Fluit AC. Whole genome analysis of a livestock-associated methicillin-resistant Staphylococcus aureus ST398 isolate from a case of human endocarditis. BMC Genomics. 2010 Jun;11(1):376.

12. Frampton M, Houlston R. Generation of Artificial FASTQ Files to Evaluate the Performance of Next-Generation Sequencing Pipelines. Badger JH, editor. PLoS ONE. 2012 Nov;7(11):e49110.

13. Croucher NJ, Page AJ, Connor TR, Delaney AJ, Keane JA, Bentley SD, et al. Rapid phylogenetic analysis of large samples of recombinant bacterial whole genome sequences using Gubbins. Nucleic Acids Research. 2015 Feb;43(3):e15.

14. Didelot X, Wilson DJ. ClonalFrameML: Efficient Inference of Recombination in Whole Bacterial Genomes. PLoS Computational Biology. 2015;11(2):e1004041.

15. Price LB, Stegger M, Hasman H, Aziz M, Larsen J, Andersen PS, et al. *Staphylococcus aureus* CC398: Host adaptation and emergence of methicillin resistance in livestock. mBio. 2012 Mar;3(1):1–6.

16. Stamatakis A. RAxML version 8: a tool for phylogenetic analysis and post-analysis of large phylogenies. Bioinformatics. 2014 May 1;30(9):1312–3.

17. Letunic I, Bork P. Interactive Tree Of Life (iTOL) v5: an online tool for phylogenetic tree display and annotation. Nucleic Acids Research. 2021 Jul 2;49(W1):W293–6.

18. Seemann T. Prokka: Rapid prokaryotic genome annotation. Bioinformatics. 2014 Jul;30(14):2068–9.

19. Tonkin-Hill G, MacAlasdair N, Ruis C, Weimann A, Horesh G, Lees JA, et al. Producing polished prokaryotic pangenomes with the Panaroo pipeline. Genome Biol. 2020 Jul;21(1):1–21.

20. Paradis E, Schliep K. ape 5.0: an environment for modern phylogenetics and evolutionary analyses in R. Bioinformatics. 2019 Feb 1;35(3):526–8.

21. Jombart T, Kendall M, Almagro-Garcia J, Colijn C. treespace: Statistical exploration of landscapes of phylogenetic trees. Molecular Ecology Resources. 2017 Nov;17(6):1385–92.

22. Kendall M, Colijn C. Mapping Phylogenetic Trees to Reveal Distinct Patterns of Evolution. Molecular Biology and Evolution. 2016 Oct;33(10):2735–43.

23. Shapiro B, Rambaut A, Drummond AJ. Choosing appropriate substitution models for the phylogenetic analysis of  protein-coding sequences. Vol. 23, Molecular biology and evolution. United States; 2006. p. 7–9.

24. Rambaut A, Suchard M, Xie D, Drummond A. Tracer v1.6. 2014.
