# Supplementary material for: The host phylogeny determines viral infectivity and replication across Staphylococcus host species
Source: PLoS Pathog. 2023 Jun 8;19(6):e1011433. doi: 10.1371/journal.ppat.1011433 (PMC10284401; doi:10.1371/journal.ppat.1011433)
Supplement: S1 Table — Clonal complex and sequence type, where available, were determined using PubMLST [23]. S. aureus strains missing a ST are coagulase negative and therefore do not have an MLST scheme and the S. aureus strains with STs but no CCs are not similar enough to other strains to be assigned a CC. (DOCX) [file ppat.1011433.s002.docx]

**S1 Table:** ***Staphylococcaceae* samples metadata.** Clonal complex (CC) and sequence type (ST), where available, were determined using PubMLST (23). *S. aureus* strains missing a ST are coagulase negative and therefore do not have an MLST scheme and the *S. aureus* strains with STs but no CCs are not similar enough to other strains to be assigned a CC.

| **Isolate ID** | **Species** | **Host** | **Location** | **Year** | **Clonal Complex** | **Sequence Type** | **NCBI BioSample ID** | **Obtained from** |
| --- | --- | --- | --- | --- | --- | --- | --- | --- |
| 13S44S9 | *S. aureus* | *Homo sapiens* | BEL | 2012 | CC8 | ST-8 | SAMN31484028 | Jean-Paul Pirnay |
| 271Y | *S. kloosii* | *Eptesicus serotinus* | GBR | 2015 | --- | --- | SAMN31484029 | Edward Feil |
| 2111F7LW | *M. sciuri* | *Pteropus livingstonii* | GBR | 2017 | --- | --- | SAMN31484030 | Edward Feil |
| 27420LC | *S. simiae* | *Pteropus livingstonii* | GBR | 2016 | --- | --- | SAMN31484031 | Edward Feil |
| 2745SW | *S. nepalensis* | *Pteropus livingstonii* | GBR | 2016 | --- | --- | SAMN31484032 | Edward Feil |
| 82B | *S. caeli* | Environmental | ITA | 2013 | --- | --- | SAMEA2297795 | Gavin Paterson |
| 8325-4 | *S. aureus* | *---* | --- | --- | CC8 | ST-8 | SAMN31484033 | Edward Feil |
| AR03918O1 | *S. aureus* | *Sciurus carolinensis* | GBR | 2018 | --- | ST-133 | SAMN31484034 | Edward Feil |
| AR05S1 | *S. aureus* | *Sciurus carolinensis* | GBR | 2015 | --- | --- | SAMN31484035 | Edward Feil |
| AR05618O1 | *S. aureus* | *Sciurus carolinensis* | GBR | 2018 | --- | ST-49 | SAMN31484036 | Edward Feil |
| ASARM61 | *S. aureus* | *Homo sapiens* | GBR | 2006 | CC22 | ST-22 | SAMN31484037 | Edward Feil |
| ASARM70 | *S. aureus* | *Homo sapiens* | GBR | 2006 | CC22 | ST-22 | SAMN31484038 | Edward Feil |
| ASARM71 | *S. aureus* | *Homo sapiens* | GBR | 2006 | CC22 | ST-22 | SAMN31484039 | Edward Feil |
| ASARM72 | *S. aureus* | *Homo sapiens* | GBR | 2006 | CC22 | ST-22 | SAMN31484040 | Edward Feil |
| ASARM73 | *S. aureus* | *Homo sapiens* | GBR | 2006 | CC22 | ST-22 | SAMN31484041 | Edward Feil |
| ASARM74 | *S. aureus* | *Homo sapiens* | GBR | 2006 | CC22 | ST-22 | SAMN31484042 | Edward Feil |
| NCTC7692 | *S. saprophyticus subsp. saprophyticus* | Environmental | --- | 1948 | --- | --- | SAMEA3517999 | Gavin Paterson |
| NCTC11320 | *S. hominis spp hominis* | *Homo sapiens* | USA | 1975 | --- | --- | SAMEA3539708 | Gavin Paterson |
| NCTC11043 | *S. xylosus* | *Homo sapiens* | USA | 1975 | --- | --- | SAMEA3539705 | Gavin Paterson |
| B128S3 | *S. aureus* | *Sciurus carolinensis* | GBR | 2015 | CC1 | ST-188 | SAMN31484043 | Edward Feil |
| B142S1 | *S. aureus* | *Sciurus carolinensis* | GBR | 2015 | --- | ST-692 | SAMN31484044 | Edward Feil |
| DAR04181C1 | *S. aureus* | *Cervus elaphus* | GBR | 2018 | CC8 | ST-1958 | SAMN31484045 | Edward Feil |
| DAR06181LC1 | *S. aureus* | *Cervus elaphus* | GBR | 2018 | --- | ST-3237 | SAMN31484046 | Edward Feil |
| DAR091813 | *S. aureus* | *Cervus elaphus* | GBR | 2018 | --- | ST-425 | SAMN31484047 | Edward Feil |
| DEU1 | *S. aureus* | *Homo sapiens* | TUR | 2009 | CC8 | ST-239 | SAMN31484048 | Edward Feil |
| DEU2 | *S. aureus* | *Homo sapiens* | TUR | 2009 | CC8 | ST-239 | SAMN31484049 | Edward Feil |
| DSM104441 | *S. edaphicus* | Environmental | ATA | 2013 | --- | --- | SAMN31484050 | Gavin Paterson |
| DSM107950 | *S. pseudoxylosus* | *Bos taurus* | FRA | 2002 | --- | --- | SAMN31484051 | Gavin Paterson |
| DSM18669 | *S. saprophyticus subsp. Bovis* | *Bos taurus* | CZE | 1996 | --- | --- | SAMN31484052 | Gavin Paterson |
| DSM21284 | *S. pseudointermedius* | *Felis catus* | BEL | 2008 | --- | --- | SAMN31484053 | Gavin Paterson |
| NCTC12218 | *S. schleiferi subsp. coagulans* | *Homo sapiens* | --- | 1988 | --- | --- | SAMEA3221103 | Gavin Paterson |
| DSM6628 | *S. schleiferi subsp. schleiferi* | *Canis lupus* | --- | 1991 | --- | --- | SAMN31484054 | Gavin Paterson |
| EOE23 | *S. aureus* | *Homo sapiens* | GBR | 1998 | CC30 | ST-36 | SAMN31484055 | Edward Feil |
| EOE03 | *S. aureus* | *Homo sapiens* | GBR | 1998 | CC30 | ST-3488 | SAMN31484056 | Edward Feil |
| EOE30 | *S. aureus* | *Homo sapiens* | GBR | 1998 | CC30 | ST-36 | SAMN31484057 | Edward Feil |
| EOE35 | *S. aureus* | *Homo sapiens* | GBR | 2003 | CC30 | ST-36 | SAMN31484058 | Edward Feil |
| EOE41 | *S. aureus* | *Homo sapiens* | GBR | 2005 | CC30 | ST-36 | SAMN31484059 | Edward Feil |
| EOE42 | *S. aureus* | *Homo sapiens* | GBR | 2005 | CC30 | ST-36 | SAMN31484060 | Edward Feil |
| HU25 | *S. aureus* | *Homo sapiens* | BRA | 1905 | CC8 | ST-239 | SAMN31484061 | Edward Feil |
| JW32660O5 | *S. aureus* | *Sciurus carolinensis* | GBR | 2018 | --- | ST-49 | SAMN31484062 | Edward Feil |
| JW30866OBHY3 | *S. aureus* | *Sciurus carolinensis* | GBR | 2018 | --- | ST-49 | SAMN31484063 | Edward Feil |
| JW31330LBHY2 | *S. aureus* | *Sciurus carolinensis* | GBR | 2018 | --- | ST-49 | SAMN31484064 | Edward Feil |
| JW31330OBHY1 | *S. aureus* | *Sciurus carolinensis* | GBR | 2018 | --- | ST-49 | SAMN31484065 | Edward Feil |
| MU1 | *S. aureus* | *Homo sapiens* | TUR | 2010 | CC8 | --- | SAMN31484066 | Edward Feil |
| MU2 | *S. aureus* | *Homo sapiens* | TUR | 2010 | CC8 | ST-239 | SAMN31484067 | Edward Feil |
| NCTC11042 | *S. haemolyticus* | *Homo sapiens* | CZE | 1976 | --- | --- | SAMEA3233544 | Gavin Paterson |
| NCTC11046 | *S. simulans* | *Homo sapiens* | CZE | 1976 | --- | --- | SAMEA3504572 | Gavin Paterson |
| NCTC11047 | *S. epidermidis* | *Homo sapiens* | CZE | 1976 | --- | ST-5 | SAMEA3233545 | Gavin Paterson |
| P32 | *S. aureus* | *Homo sapiens* | POL | 1996 | CC8 | ST-239 | SAMN31484068 | Edward Feil |
| SaTPS3026 | *S. aureus* | *Homo sapiens* | AUS | 2013 | CC93 | ST-93 | SAMN31484069 | Edward Feil |
| SaTPS3043 | *S. aureus* | *Homo sapiens* | AUS | 2013 | CC30 | ST-30 | SAMN31484070 | Edward Feil |
| SaTPS3072 | *S. aureus* | *Homo sapiens* | AUS | 2013 | CC1 | ST-1 | SAMN31484071 | Edward Feil |
| SaTPS3097 | *S. aureus* | *Homo sapiens* | AUS | 2013 | CC8 | ST-8 | SAMN31484072 | Edward Feil |
| SaTPS3104 | *S. aureus* | *Homo sapiens* | AUS | 2013 | CC93 | ST-93 | SAMN31484073 | Edward Feil |
| SaTPS3105 | *S. aureus* | *Homo sapiens* | AUS | 2013 | CC93 | CT-93 | SAMN31484074 | Edward Feil |
| SAR1018S1 | *S. aureus* | *Ovis aries* | GBR | 2018 | CC8 | ST-8 | SAMN31484075 | Edward Feil |
| SAR1218N1 | *S. aureus* | *Ovis aries* | GBR | 2018 | --- | ST-1640 | SAMN31484076 | Edward Feil |
| SAR1418N1 | *S. aureus* | *Ovis aries* | GBR | 2018 | --- | ST-130 | SAMN31484077 | Edward Feil |
| USFL008 | *S. aureus* | *Homo sapiens* | USA | 2009 | CC8 | ST-8 | SAMN31484078 | Edward Feil |
| USFL009 | *S. aureus* | *Homo sapiens* | USA | 2009 | CC8 | ST-8 | SAMN31484079 | Edward Feil |
| USFL012 | *S. aureus* | *Homo sapiens* | USA | 2009 | CC8 | ST-8 | SAMN31484080 | Edward Feil |
| USFL016 | *S. aureus* | *Homo sapiens* | USA | 2009 | CC8 | ST-8 | SAMN31484081 | Edward Feil |
| USFL018 | *S. aureus* | *Homo sapiens* | USA | 2009 | CC8 | ST-8 | SAMN31484082 | Edward Feil |
| USFL020 | *S. aureus* | *Homo sapiens* | USA | 2009 | CC8 | ST-8 | SAMN31484083 | Edward Feil |
