# Supplementary material for: The host phylogeny determines viral infectivity and replication across Staphylococcus host species
Source: PLoS Pathog. 2023 Jun 8;19(6):e1011433. doi: 10.1371/journal.ppat.1011433 (PMC10284401; doi:10.1371/journal.ppat.1011433)
Supplement: S2 Table — Estimates of repeatability are taken from model (2) and estimates of phylogenetic heritability (the proportion of phylogenetic and non-phylogenetic strain variation explained by the host phylogeny) and phylogenetic heritability of the total variance (the proportion of total variation explained by the host phylogeny) are taken from model (1). PA = plaque assay, CI = credible interval. (DOCX) [file ppat.1011433.s003.docx]

**S2 Table:** **Estimates for the repeatability and heritability across 64 *Staphylococcaceae*** **isolates when no outliers were removed from the OD data.** Estimates of repeatability are taken from model (2) and estimates of phylogenetic heritability (the proportion of phylogenetic and non-phylogenetic strain variation explained by the host phylogeny) and phylogenetic heritability of the total variance (the proportion of total variation explained by the host phylogeny) are taken from model (1). PA = plaque assay, CI = credible interval.

|  | **Repeatability** | | **Phylogenetic heritability** | | **Phylogenetic heritability of total variance** | |  |
| --- | --- | --- | --- | --- | --- | --- | --- |
| **Method** | Mean | 95% CI | Mean | 95% CI | Mean | 95% CI | |
| **Binary PA** | 1.00 | 1.00, 1.00 | 1.00 | 1.00, 1.00 | 1.00 | 1.00, 1.00 | |
| **Continuous PA** | 0.00 | 0.00, 0.00 | 0.65 | 0.01, 1.00 | 0.00 | 0.00, 0.00 | |
| **OD** | 0.94 | 0.89, 0.98 | 0.99 | 0.96, 1.00 | 0.91 | 0.79, 0.98 | |
| **qPCR** | 0.99 | 0.99, 0.99 | 1.00 | 1.00, 1.00 | 0.99 | 0.99, 1.00 | |
