# Supplementary material for: The host phylogeny determines viral infectivity and replication across Staphylococcus host species
Source: PLoS Pathog. 2023 Jun 8;19(6):e1011433. doi: 10.1371/journal.ppat.1011433 (PMC10284401; doi:10.1371/journal.ppat.1011433)
Supplement: S5 Table — Numbers show the mean estimates for the correlation strength (r, white cells) and slope (β, grey cells) between pairs of methods, with 95% credible intervals (CIs) indicated in brackets. The slopes were calculated with columns as x and rows as y. Estimates with CIs that do not span zero are highlighted in bold. PA = plaque assay, *value on a probit scale. (DOCX) [file ppat.1011433.s006.docx]

**S5 Table: Estimates from the phylogenetic generalised linear mixed models for inter-strain correlations in susceptibility between methods where major outliers were removed from the OD data.** Numbers show the mean estimates for the correlation strength (r, white cells) and slope (β, grey cells) between pairs of methods, with 95% credible intervals (CIs) indicated in brackets. The slopes were calculated with columns as x and rows as y. Estimates with CIs that do not span zero are highlighted in bold. PA = plaque assay, *value on a probit scale.

|  | **Binary PA** | **Continuous PA** | **OD** | **qPCR** |
| --- | --- | --- | --- | --- |
| **Binary PA** | - | - | **0.89**  **(0.75, 0.98)** | **0.97**  **(0.91, 1.00)** |
| **Continuous PA** | - | - | -0.00  (-0.97, 0.97) | -0.01  (-0.99, 1.00) |
| **OD** | **0.50***  **(0.50, 0.50)** | 0.02  (-0.33, 0.30) | - | **0.92**  **(0.83, 0.98)** |
| **qPCR** | **0.51***  **(0.51, 0.52)** | 0.23  (-1.77, 1.45) | **4.67**  **(3.20, 6.35)** | - |
