# Supplementary material for: The host phylogeny determines viral infectivity and replication across Staphylococcus host species
Source: PLoS Pathog. 2023 Jun 8;19(6):e1011433. doi: 10.1371/journal.ppat.1011433 (PMC10284401; doi:10.1371/journal.ppat.1011433)
Supplement: S6 Table — (DOCX) [file ppat.1011433.s007.docx]

**S6 Table:** ***Staphylococcaceae* assembly QC**.

| **Isolate ID** | **Species** | **Total sequence length** | **Number of Contigs** | **N50** | **N75** | **L50** | **L75** | **CG (%)** |
| --- | --- | --- | --- | --- | --- | --- | --- | --- |
| 13S44S9 | *S. aureus* | 2689205 | 48 | 537826 | 305075 | 2 | 3 | 33.74 |
| 271Y | *S. kloosii* | 2792890 | 111 | 422410 | 297912 | 2 | 4 | 33.59 |
| 2111F7LW | *M. sciuri* | 2791973 | 10 | 1626732 | 686595 | 1 | 2 | 32.6 |
| 27420LC | *S. simiae* | 2872001 | 591 | 60836 | 24834 | 17 | 35 | 39.65 |
| 2745SW | *S. nepalensis* | 2990883 | 88 | 432000 | 225788 | 3 | 5 | 34.26 |
| 8325-4 | *S. aureus* | 2667871 | 15 | 545622 | 305337 | 2 | 3 | 32.79 |
| AR03918O1 | *S. aureus* | 2715370 | 49 | 289866 | 159650 | 4 | 7 | 33.8 |
| AR05S1 | *S. aureus* | 2730240 | 6 | 1400027 | 1029280 | 1 | 2 | 32.89 |
| AR05618O1 | *S. aureus* | 2754645 | 26 | 201219 | 110286 | 3 | 8 | 32.87 |
| ASARM61 | *S. aureus* | 2844676 | 47 | 218124 | 130308 | 5 | 9 | 33.12 |
| ASARM70 | *S. aureus* | 2793704 | 31 | 253974 | 125498 | 3 | 8 | 32.95 |
| ASARM71 | *S. aureus* | 2913732 | 40 | 178141 | 109415 | 6 | 11 | 32.93 |
| ASARM72 | *S. aureus* | 2927497 | 58 | 177011 | 112848 | 6 | 11 | 33.4 |
| ASARM73 | *S. aureus* | 2751546 | 68 | 176649 | 93924 | 6 | 12 | 33.32 |
| ASARM74 | *S. aureus* | 2793305 | 38 | 212016 | 130308 | 5 | 9 | 33.16 |
| B128S3 | *S. aureus* | 2749860 | 25 | 754935 | 587839 | 2 | 3 | 33.17 |
| B142S1 | *S. aureus* | 2771562 | 20 | 506681 | 174678 | 2 | 5 | 33.13 |
| DAR04181C1 | *S. aureus* | 2778159 | 23 | 365053 | 186399 | 2 | 5 | 33.01 |
| DAR06181LC1 | *S. aureus* | 2767305 | 50 | 457290 | 177150 | 3 | 6 | 33.44 |
| DAR091813 | *S. aureus* | 2741212 | 41 | 894575 | 894575 | 2 | 2 | 33.4 |
| DEU1 | *S. aureus* | 2877934 | 54 | 195490 | 76753 | 6 | 14 | 32.75 |
| DEU2 | *S. aureus* | 2900536 | 69 | 177555 | 64149 | 6 | 14 | 32.93 |
| DSM104441 | *S. edaphicus* | 2705388 | 57 | 111576 | 79623 | 8 | 15 | 33.95 |
| DSM107950 | *S. pseudoxylosus* | 3015572 | 15 | 744489 | 444013 | 2 | 4 | 32.82 |
| DSM18669 | *S. saprophyticus subsp. Bovis* | 2683087 | 43 | 177515 | 92876 | 4 | 10 | 33.23 |
| DSM21284 | *S. pseudointermedius* | 2566130 | 123 | 171890 | 80459 | 6 | 12 | 39.42 |
| DSM6628 | *S. schleiferi subsp. schleiferi* | 2482579 | 50 | 99751 | 57037 | 9 | 16 | 35.92 |
| EOE23 | *S. aureus* | 2891105 | 62 | 152503 | 85263 | 7 | 12 | 33.07 |
| EOE03 | *S. aureus* | 2918284 | 80 | 174945 | 98277 | 6 | 11 | 33.58 |
| EOE30 | *S. aureus* | 2957023 | 107 | 152504 | 85211 | 7 | 12 | 33.89 |
| EOE35 | *S. aureus* | 2922392 | 63 | 170766 | 127397 | 6 | 11 | 33.34 |
| EOE41 | *S. aureus* | 3142949 | 388 | 171014 | 68838 | 6 | 13 | 37.77 |
| EOE42 | *S. aureus* | 2896607 | 84 | 174997 | 150473 | 6 | 10 | 33.75 |
| HU25 | *S. aureus* | 3044558 | 235 | 111559 | 64770 | 9 | 18 | 35.57 |
| JW32660O5 | *S. aureus* | 2726977 | 16 | 252367 | 168282 | 3 | 6 | 32.97 |
| JW30866OBHY3 | *S. aureus* | 2689832 | 22 | 272042 | 121404 | 3 | 7 | 32.84 |
| JW31330LBHY2 | *S. aureus* | 2655130 | 31 | 661610 | 128617 | 2 | 5 | 33.18 |
| JW31330OBHY1 | *S. aureus* | 2692873 | 21 | 661610 | 168286 | 2 | 5 | 33.02 |
| MU1 | *S. aureus* | 2925846 | 83 | 109489 | 46141 | 8 | 19 | 31.92 |
| MU2 | *S. aureus* | 2899275 | 115 | 96721 | 52779 | 8 | 18 | 33.81 |
| P32 | *S. aureus* | 3052463 | 172 | 181350 | 87811 | 6 | 13 | 34.96 |
| SaTPS3026 | *S. aureus* | 2803251 | 19 | 647590 | 116190 | 2 | 6 | 32.8 |
| SaTPS3043 | *S. aureus* | 2917497 | 204 | 314036 | 137550 | 3 | 7 | 35.92 |
| SaTPS3072 | *S. aureus* | 2831386 | 24 | 762256 | 762256 | 2 | 2 | 33.13 |
| SaTPS3097 | *S. aureus* | 2908175 | 35 | 488593 | 120006 | 2 | 5 | 32.92 |
| SaTPS3104 | *S. aureus* | 2813776 | 32 | 297283 | 167711 | 3 | 6 | 33.03 |
| SaTPS3105 | *S. aureus* | 2998415 | 385 | 260707 | 100823 | 4 | 9 | 38.03 |
| SAR1018S1 | *S. aureus* | 2732835 | 28 | 297495 | 141848 | 3 | 6 | 32.99 |
| SAR1218N1 | *S. aureus* | 2807838 | 38 | 262488 | 137087 | 4 | 8 | 32.69 |
| SAR1418N1 | *S. aureus* | 2796391 | 16 | 920935 | 186803 | 2 | 4 | 32.74 |
| USFL008 | *S. aureus* | 2938818 | 137 | 474662 | 173146 | 2 | 5 | 34.67 |
| USFL009 | *S. aureus* | 2882700 | 30 | 488618 | 332886 | 2 | 4 | 32.78 |
| USFL012 | *S. aureus* | 2920357 | 65 | 381147 | 110994 | 3 | 6 | 33.54 |
| USFL016 | *S. aureus* | 2913175 | 73 | 867764 | 345560 | 2 | 3 | 33.87 |
| USFL018 | *S. aureus* | 2881247 | 28 | 399500 | 332899 | 3 | 5 | 32.9 |
| USFL020 | *S. aureus* | 2938572 | 155 | 345560 | 139318 | 3 | 6 | 34.93 |
