# Supplementary material for: The host phylogeny determines viral infectivity and replication across Staphylococcus host species
Source: PLoS Pathog. 2023 Jun 8;19(6):e1011433. doi: 10.1371/journal.ppat.1011433 (PMC10284401; doi:10.1371/journal.ppat.1011433)
Supplement: S7 Table — (DOCX) [file ppat.1011433.s008.docx]

# **S7 Table: *Staphylococcaceae*** **sequences used in the FastQ Screen to determine if all sequences used in this study belong to *Staphylococcaceae*.**

| **Species** | **Strain** | **GenBank Accession Number** |
| --- | --- | --- |
| S. aureus | LGA251 | GCA_000237265.1 |
| S. caeli | 82B | GCA_900097965.1 |
| S. capitis | AYP1020 | GCA_001028645.1 |
| S. cohnii | SNUDS-2 | GCA_001990205.1 |
| S. condimenti | DSM_11674 | GCA_001922405.1 |
| S. edaphicus | CM_8730 | GCF_002614725.1 |
| S. epidermidis | ATCC_12228 | GCA_000007645.1 |
| S. haemolyticus | JCSC1435 | GCA_000009865.1 |
| S. hominis | K1 | GCA_002850375.1 |
| S. hyicus | ATCC_11249 | GCA_000816085.1 |
| S. kloosii | CNV2_Masurca_598 | GCA_001593625.1 |
| S. nepalensis | JS1 | GCA_002442895.1 |
| S. pasteuri | SP1 | GCA_000494875.1 |
| S. pettenkoferi | FDAARGOS_288 | GCA_002208805.2 |
| S. pseudintermedius | HKU10-03 | GCA_000185885.1 |
| S. saprophyticus | ATCC_15305 | GCA_007814115.1 |
| M. sciuri | FDAARGOS_285 | GCA_002209165.2 |
| S. simiae | NCTC13838 | GCA_900187055.1 |
| S. simulans | FDAARGOS_124 | GCA_001559115.2 |
| S. stepanovicii | NCTC13839 | GCA_900187075.1 |
| S. succinus | 14BME20 | GCF_001902315.1 |
| S. warneri | SG1 | GCA_007668085.1 |
| S. xylosus | HKUOPL8 | GCA_000706685.1 |
