# Supplementary material for: The host phylogeny determines viral infectivity and replication across Staphylococcus host species
Source: PLoS Pathog. 2023 Jun 8;19(6):e1011433. doi: 10.1371/journal.ppat.1011433 (PMC10284401; doi:10.1371/journal.ppat.1011433)
Supplement: S10 Table — Numbers show the mean estimates for the correlation strength (r, white cells) and slope (β, grey cells) between pairs of methods, with 95% credible intervals (CIs) indicated in brackets. The slopes were calculated with columns as x and rows as y. Estimates with CIs that do not span zero are highlighted in bold. PA = plaque assay, *value on a probit scale. (DOCX) [file ppat.1011433.s011.docx]

# **S10 Table: Inter-strain correlations between methods for assessing host range in a within-*aureus* model.** Numbers show the mean estimates for the correlation strength (r, white cells) and slope (β, grey cells) between pairs of methods, with 95% credible intervals (CIs) indicated in brackets. The slopes were calculated with columns as x and rows as y. Estimates with CIs that do not span zero are highlighted in bold. PA = plaque assay, *value on a probit scale.

|  | **Binary PA** | **Continuous PA** | **OD** | **qPCR** |
| --- | --- | --- | --- | --- |
| **Binary PA** | - | - | -0.12  (-0.67, 0.48) | -0.67  (-0.39, 0.82) |
| **Continuous PA** | - | - | 0.02  (-0.94, 0.91) | 0.03  (-0.91, 0.95) |
| **OD** | **0.50***  **(0.49, 0.51)** | 0.03  (-0.81, 0.86) | - | **0.77**  **(0.47, 0.96)** |
| **qPCR** | **0.51***  **(0.49, 0.53)** | -0.24  (-2.19, 1.94) | **1.74**  **(0.53, 3.02)** | - |
