# Supplementary material for: The host phylogeny determines viral infectivity and replication across Staphylococcus host species
Source: PLoS Pathog. 2023 Jun 8;19(6):e1011433. doi: 10.1371/journal.ppat.1011433 (PMC10284401; doi:10.1371/journal.ppat.1011433)
Supplement: S11 Table — Numbers show the mean estimates for the correlation strength (r, white cells) and slope (β, grey cells) between pairs of methods, with 95% credible intervals (CIs) indicated in brackets. The slopes were calculated with columns as x and rows as y. Estimates with CIs that do not span zero are highlighted in bold. PA = plaque assay, *value on a probit scale. (DOCX) [file ppat.1011433.s012.docx]

# **S11 Table: Inter-species correlations between methods for assessing host range in a between-species model.** Numbers show the mean estimates for the correlation strength (r, white cells) and slope (β, grey cells) between pairs of methods, with 95% credible intervals (CIs) indicated in brackets. The slopes were calculated with columns as x and rows as y. Estimates with CIs that do not span zero are highlighted in bold. PA = plaque assay, *value on a probit scale.

|  | **Binary PA** | **Continuous PA** | **OD** | **qPCR** |
| --- | --- | --- | --- | --- |
| **Binary PA** | - | - | **0.79**  **(0.46, 0.99)** | **0.86**  **(0.62, 1.00)** |
| **Continuous PA** | - | - | 0.03  (-0.96, 0.99) | 0.03  (-0.99, 0.98) |
| **OD** | **0.50***  **(0.50, 0.51)** | -0.00  (-0.09, 0.07) | - | **0.89**  **(0.72, 1.00)** |
| **qPCR** | **0.52***  **(0.50, 0.56)** | -0.02  (-0.57, 0.43) | **5.92**  **(3.47, 8.39)** | - |
