# Supplementary material for: The host phylogeny determines viral infectivity and replication across Staphylococcus host species
Source: PLoS Pathog. 2023 Jun 8;19(6):e1011433. doi: 10.1371/journal.ppat.1011433 (PMC10284401; doi:10.1371/journal.ppat.1011433)
Supplement: S1 Fig — MDS visualization of tree distances when λ = 0. As the projection often requires that multiple trees are plotted at the same co-ordinates, contour lines are used to indicate the density of points. (DOCX) [file ppat.1011433.s013.docx]

**S1 Fig: A comparison of all 123 trees on 64 tips using the Kendall Colijn metric vector (mid-point rooted gene trees).** MDS visualization of tree distances when λ = 0. As the projection often requires that multiple trees are plotted at the same co-ordinates, contour lines are used to indicate the density of points.
