# Supplementary material for: The host phylogeny determines viral infectivity and replication across Staphylococcus host species
Source: PLoS Pathog. 2023 Jun 8;19(6):e1011433. doi: 10.1371/journal.ppat.1011433 (PMC10284401; doi:10.1371/journal.ppat.1011433)
Supplement: S2 Fig — (DOCX) [file ppat.1011433.s014.docx]

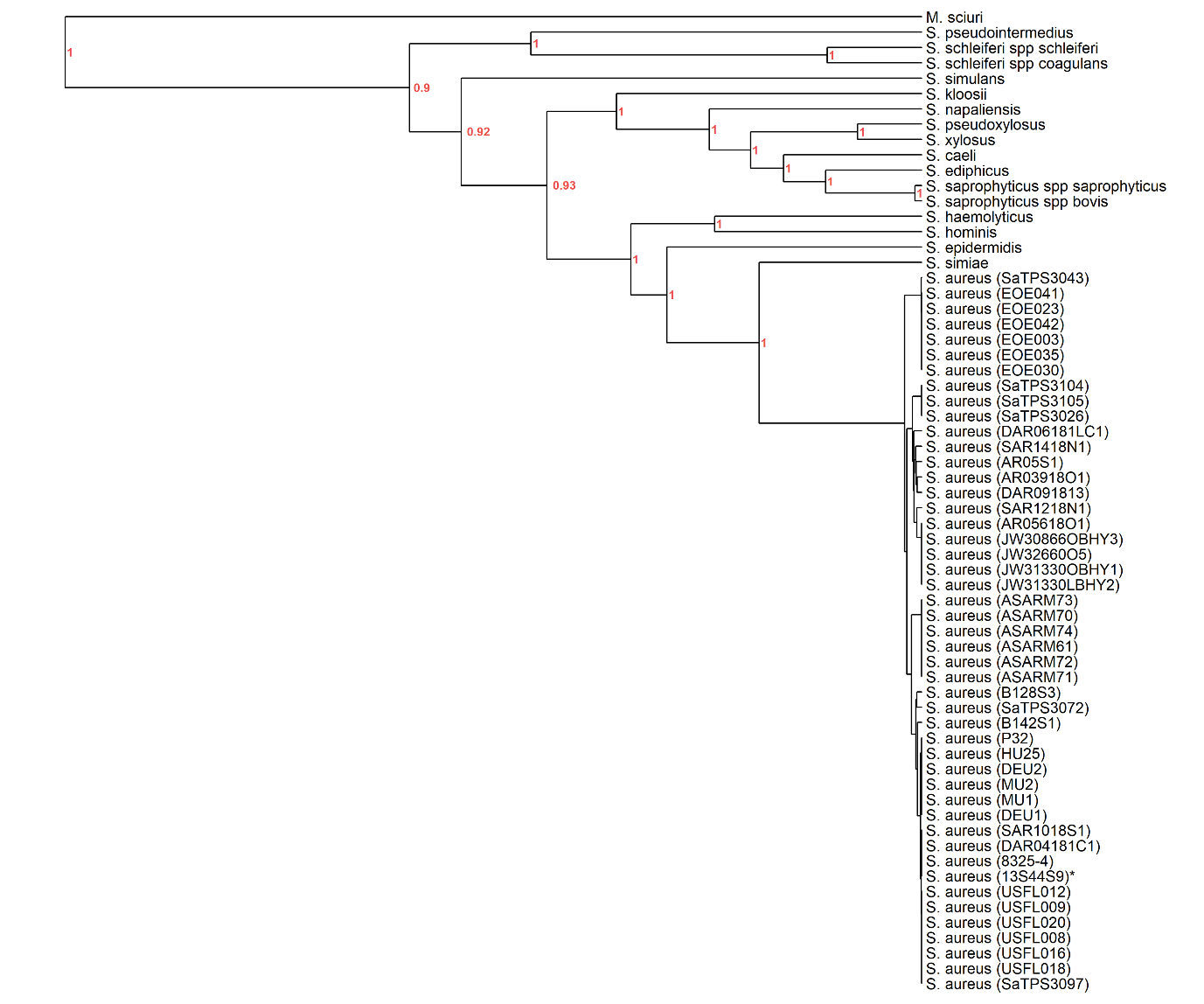


**S2 Fig: Core genome phylogeny of 64 *Staphylococcaceae* samples with the posterior probabilities of the MCMC chain displayed.**
