# Supplementary material for: The host phylogeny determines viral infectivity and replication across Staphylococcus host species
Source: PLoS Pathog. 2023 Jun 8;19(6):e1011433. doi: 10.1371/journal.ppat.1011433 (PMC10284401; doi:10.1371/journal.ppat.1011433)
Supplement: S4 Fig — (DOCX) [file ppat.1011433.s016.docx]

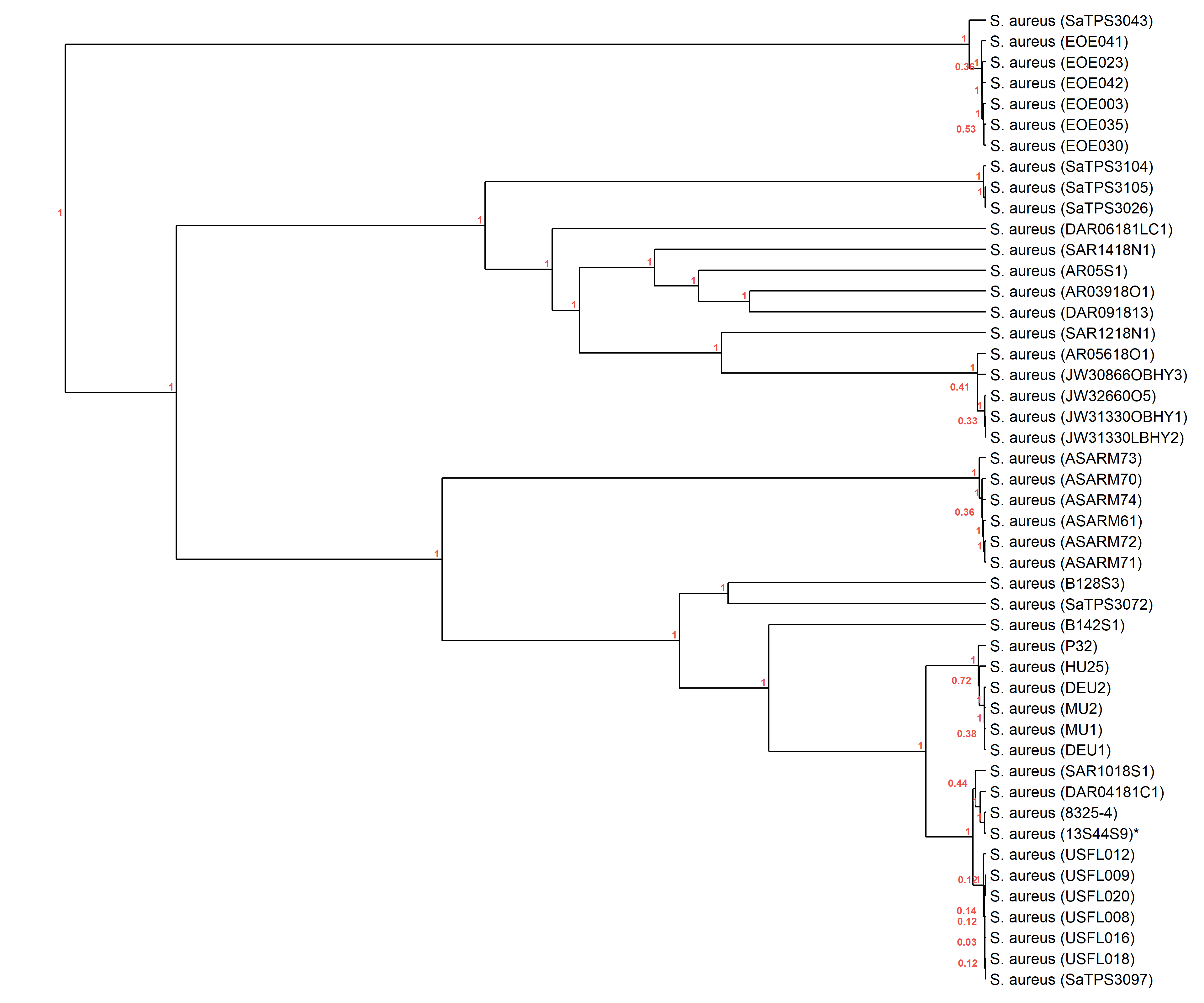


**S4 Fig: A subset of the core genome phylogeny showing 47 *Staphylococcus aureus* samples with the posterior probabilities of the MCMC chain displayed.**
